# Lipidomic Analysis of Liver and Adipose Tissue in a High-Fat Diet-Induced Non-Alcoholic Fatty Liver Disease Mice Model Reveals Alterations in Lipid Metabolism by Weight Loss and Aerobic Exercise

**DOI:** 10.3390/molecules29071494

**Published:** 2024-03-27

**Authors:** Thomai Mouskeftara, Olga Deda, Grigorios Papadopoulos, Antonios Chatzigeorgiou, Helen Gika

**Affiliations:** 1 Laboratory of Forensic Medicine & Toxicology, Department of Medicine, Aristotle University of Thessaloniki, 54124 Thessaloniki, Greece; mousthom@auth.gr (T.M.); oliadmy@gmail.com (O.D.); 2 Biomic AUTh, Center for Interdisciplinary Research and Innovation (CIRI-AUTH), Balkan Center B1.4, 10th km Thessaloniki-Thermi Rd, P.O. Box 8318, 57001 Thessaloniki, Greece; 3 Department of Physiology, Medical School, National and Kapodistrian University of Athens, 75 Mikras Asias Str., 11527 Athens, Greece; grpapad@hotmail.com (G.P.); achatzig@med.uoa.gr (A.C.)

**Keywords:** non-alcoholic fatty liver disease, exercise, de novo lipogenesis, lipidomics, liver, adipose tissue

## Abstract

Detailed investigation of the lipidome remodeling upon normal weight conditions, obesity, or weight loss, as well as the influence of physical activity, can help to understand the mechanisms underlying dyslipidemia in metabolic conditions correlated to the emergence and progression of non-alcoholic fatty liver disease (NAFLD). C57BL/6 male mice were fed a normal diet (ND) or a high-fat diet (HFD) for 20 weeks. Subgroups within the high-fat diet (HFD) group underwent different interventions: some engaged in exercise (HFDex), others were subjected to weight loss (WL) by changing from the HFD to ND, and some underwent a combination of weight loss and exercise (WLex) during the final 8 weeks of the 20-week feeding period. To support our understanding, not only tissue-specific lipid remodeling mechanisms but also the cross-talk between different tissues and their impact on the systemic regulation of lipid metabolism are essential. Exercise and weight loss-induced specific adaptations in the liver and visceral adipose tissue lipidomes of mice were explored by the UPLC–TOF–MS/MS untargeted lipidomics methodology. Lipidomic signatures of ND and HFD-fed mice undergoing weight loss were compared with animals with and without physical exercise. Several lipid classes were identified as contributing factors in the discrimination of the groups by multivariate analysis models, such as glycerolipids, glycerophospholipids, sphingolipids, and fatty acids, with respect to liver samples, whereas triglycerides were the only lipid class identified in visceral adipose tissue. Lipids found to be dysregulated in HFD animals are related to well-established pathways involved in the biosynthesis of PC, PE, and TG metabolism. These show a reversing trend back to basic levels of ND when animals change to a normal diet after 12 weeks, whereas the impact of exercise, though in some cases it slightly enhances the reversing trend, is not clear.

## 1. Introduction

Non-alcoholic fatty liver disease (NAFLD) has emerged as one of the most common forms of chronic liver disease and has evolved into a significant health issue worldwide [1]. Pathophysiologic hallmarks of the disease include triglyceride deposition in the liver, observed in the cytoplasm of at least 5% of hepatocytes, necrosis of hepatocytes, and inflammation [2]. According to its histological characteristics, NAFLD can be categorized as non-alcoholic fatty liver (NAFL), described by simple steatosis, and non-alcoholic steatohepatitis (NASH), which is characterized by the coexistence of steatosis, inflammation, and hepatocellular ballooning and can progress to cirrhosis, liver failure, and hepatocellular carcinoma [3].

Reviews and meta-analyses have amply demonstrated that type 2 diabetes mellitus, hyperlipidemia, metabolic syndrome, and other factors related to an unhealthy high-caloric diet are comorbid conditions commonly associated with NAFLD, or, according to the new nomenclature, metabolic dysfunction-associated steatotic liver disease (MASLD) [4,5,6,7,8,9,10]. Among all these contributors, obesity appears to play a significant role in the initial stages that lead to simple steatosis and in the progression to NASH. Obesity-induced NAFLD has been investigated in animal models applying high-fat diets (HFD) in combination with various sugars, such as fructose, which is commonly consumed in the Western world, aiming to evaluate its role in the development of NAFLD. Notably, dietary sugar intake is considered a significant mediator of hepatic steatosis and plays an essential role in the progression of NAFLD [11]. Currently, high-fructose corn syrup (HFCS), which mainly contains glucose and fructose, is the primary source of added sugars to beverages and fat-enriched diets [12]. In dysregulated liver metabolism, fructose has been proposed to affect de novo lipogenesis (DNL) by increasing the levels of enzymes involved in this process while decreasing insulin efficiency [13]. Importantly, HFCS significantly contributes to steatosis deterioration in obesity-related NAFLD, probably due to DNL upregulation in combination with tricarboxylic acid (TCA) cycle overactivation and further impairment in hepatic insulin resistance [14].

The improvement of NAFLD in obese patients can be achieved with weight loss and, more significantly, with weight loss maintenance. Studies have demonstrated that greater weight loss can lead to improvements in steatohepatitis and fibrosis [15]. In overweight patients, a 3–5% weight loss has shown a reduction in steatosis [16], while a weight loss >10% has been associated with a reduction in both steatosis and fibrosis [17]. Exercise is another effective approach for weight loss and can be utilized as part of the treatment regimen for NAFLD [18]. Notably, combining weight loss with regular exercise has been associated with improvements in transaminase levels, including a significant decrease in alanine aminotransferase (ALT) levels in patients with NASH [19]. In general, enhanced peripheral insulin sensitivity leads to a decrease in the excessive supply of free fatty acids and glucose for the synthesis of free fatty acids in the liver. Elevation in fatty acid oxidation and reduction in lipogenesis in the liver are associated with protective effects against damage to mitochondria and hepatocytes by reducing the release of damage-associated molecular patterns during exercise [5].

However, the mechanisms underlying these effects in both liver and adipose tissue have not been investigated in depth. Lipidomic studies examine the contributors to the lipid profile of the liver, but none so far have compared the changes in the hepatic and adipose tissue lipid signature triggered by weight loss and/or exercise during obesity and NAFLD. Therefore, it is important to examine the potential unidentified roles of dietary components and exercise in NAFLD pathology and its progression. In this research, we utilized a lipidomic workflow to investigate the weight loss and the effect of exercise in an obesity-induced NAFLD mice model in both hepatic and visceral adipose tissues. Our primary objective was to identify potential pathways and lipids species that might be responsible for the escalation of steatosis severity in obesity-related NAFLD mice models, particularly within the context of HFCS consumption, and explore the effect of weight loss in combination with exercise in NAFLD.

## 2. Results

### 2.1. Animal Study

**Figure 1:**
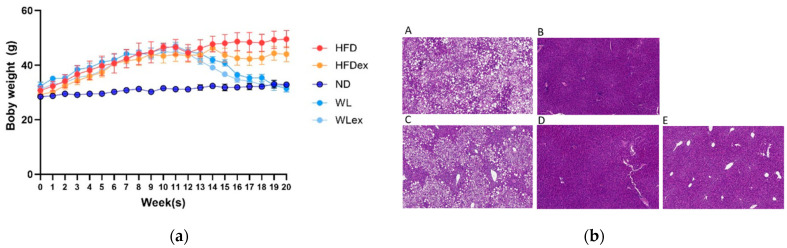
(**a**) Average (±SD) weekly body weight (g) in all mice (**b**). Representative images from Hematoxylin–Eosin (H&E)-stained liver tissue sections of (**A**) HFD, (**B**) ND, (**C**) HFDex, (**D**) WL, and (**E**) WLex mice. The *p*-values for the comparisons of body weights between HFD-HFDex, HFD-WL, HFD-WLex, ND-WL, and ND-WLex are listed in Table S1.

Many research findings have amply demonstrated that NAFLD is closely linked to metabolic imbalances associated with obesity. The examination of animal weight records indicated significant alterations primarily in relation to changes in diet, followed by exercise. Finally, body weights of weight loss (WL) and weight loss in combination with exercise (WLex) groups at the end of the 8-week intervention period were similar to the control (ND) group, whereas the HFDex group showed a decrease compared to HFD, as demonstrated in Figure 1a, where the average weekly body weight (g) (±SD) for all mice is provided. The *p*-values for the comparisons of body weights between HFD-HFDex, HFD-WL, HFD-WLex, ND-WL, and ND-WLex are listed in Table S1. According to the histological examination performed on the mouse liver samples, increased fat deposition was observed in the HFD group compared to the ND group. A reduction in hepatic steatosis was also observed in the HFDex group relative to the HFD group; however, the greatest difference was observed in the WL and WLex groups, where histological images showed remission of NAFLD, as shown in Figure 1b.

### 2.2. LC–TOF–MS Lipidomics Data

By applying the methodology for comprehensive lipid profiling, a wealth of lipid species could be detected; in liver tissue, 5298 ion signals in positive mode and 3037 in negative mode were considered after quality control filtering. Data from visceral adipose tissue were acquired only in positive ionization mode, aiming to investigate the content of glycerolipids, which ionize more effectively under this condition. The total number of corresponding ion signals reached 1092 after filtering. These data were studied by multivariate statistical methods to unravel patterns expressed by the lipidomic phenotype in the different groups. To evaluate the analytical quality of the data, in the first place, QC samples were examined (see Figures S1–S3).

**Figure 2:**
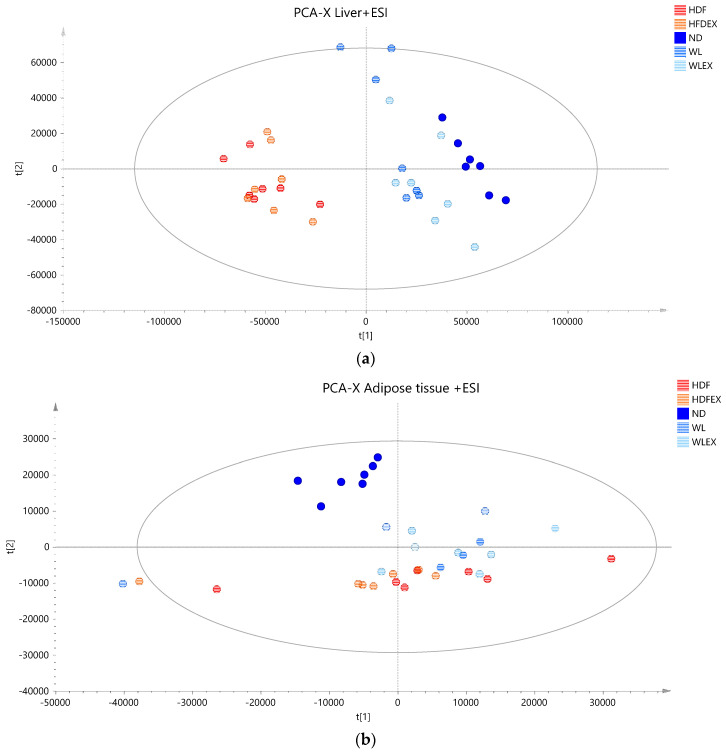

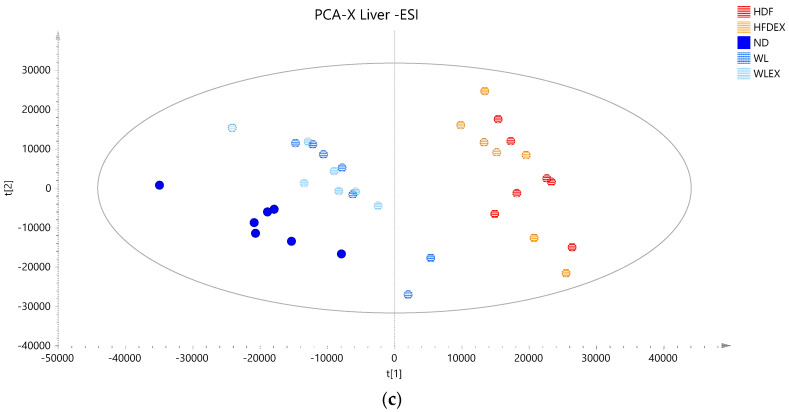
PCA score plots of mice tissue samples from the five studied groups: ND, HFD, HFDex, WL, and WLex. (**a**) Liver tissue samples projection based on positive ionization mode analysis data, (**b**) liver tissue based on negative ionization mode data, and (**c**) visceral adipose tissue samples based on positive ionization mode data. Logarithmic transformation of the data and Pareto scaling were used in all liver models in negative ionization, whereas only Pareto scaling was used in liver models in positive ionization. Logarithmic transformation of the data and Pareto scaling were used in all visceral adipose tissue models.

Based on the projection of the samples in the principal components analysis (PCA) models, it can be observed that a variation of 31% (positive ionization) and 36% (negative ionization) can be explained in the t1 axis (first component) between the hepatic tissue of mice fed with a high-fat diet (plotted in red and orange), irrespective of the parameter of exercise, and those fed with a normal diet (plotted in different color scales of blue) (Figure 2a,b). It is also clear that the two groups induced to a normal diet (WL and WLex) after the 12th week of the study exhibit greater similarity to the liver lipidomic profile of the ND mice. This finding suggests that after 8 weeks on a normal diet, lipid metabolism in the liver tends to return close to ND. This observation, however, is not reflected in the visceral adipose tissue lipidomic profile of the same mice. As can be seen in Figure 2c, weight loss groups are projected closely to HFD groups, whereas it can be concluded that ND mice have a more distinct visceral adipose tissue lipidomic profile—projected in a different quantile. This is an interesting finding that could be explained by the critical role of adipose tissue in the regulation of the systemic energy homeostasis of the organism. By using supervised models, a clearer distinction between the different groups could be observed (see PLS score plot in Figure S4), and lipid species contributing to this differentiation could be identified.

### 2.3. Hepatic Lipids Profile Reveal Alterations in Major Lipid Classes with Diet

To allow for a thorough understanding of the molecular characteristics of NAFLD associated with a high-fat diet in obesity, weight loss, and the effect of exercise, a comprehensive analysis of liver lipids was conducted. Pairwise OPLS-DA analysis between the groups revealed significant alterations in lipids between high-fat diet groups and all three normal diet mice groups (either remained—ND, either returned to normal diet—WL, WLex) independently of exercise, while no differentiation was observed when exercise was considered as the only differentiating factor (e.g., HFD vs. HFDex or WL vs. WLex). Glycerolipids, glycerophospholipids, fatty acids, and sphingolipids were found to be statistically differentiated when HFD vs. ND, HFD vs. WL, and HFD vs. WLex were compared. The quality metrics of the constructed models are presented in Table S2, whereas the detailed annotations of the statistically significant lipid species revealed from these comparisons can be found in Table S3. Table 1 summarizes all identified lipids found to be significantly altered in the liver of high-fat diet mice after weight loss or after weight loss in combination with exercise. The statistical parameters of Log2FC, *p*-values, VIP, and CV% based on the pairwise comparisons are also provided, indicating the impact on the lipids’ levels and their contributing effect on the distinct profiles that were observed.

#### 2.3.1. Hepatic Phospholipids

**Figure 3:**
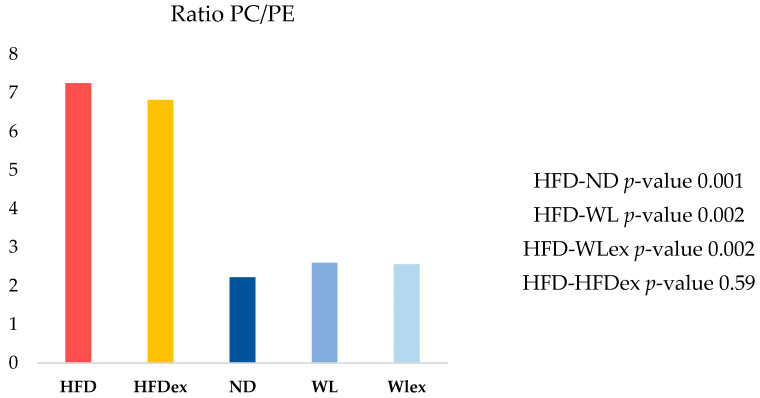
Hepatic PC/PE ratio in the five groups. A reversing trend is shown in the WL and WLex groups.

In the context of NAFLD, phosphatidylcholines (PCs) and phosphatidylethanolamines (PEs) have been linked to liver damage. In our study, we have noted statistically significant alterations in PCs, even though fold changes were relatively low between HFD mice and the other groups, as demonstrated in Table 1. On the contrary, all identified PEs, except for PE-O (38:5), exhibited substantial downregulation in the HFD group when compared to the ND, WL, and WLex groups. The ratio PC/PE was statistically significantly higher in livers from HFD compared to ND, WL, and WLex mice (Figure 3), a finding that is reported in the literature as an imbalance in the PC/PE ratio, which is linked to both fat accumulation in the liver and progression to NASH [20]. This is an indication of the reversing trend in fat accumulation in both WL groups, though no significantly enhanced impact of exercise was noted (WLex). Total phosphatidylinositol (PI) content was found to be decreased in the HFD group compared to ND, WL, and WLex mice. Lower levels of phosphatidylserine (PS) and specifically PS 38:6 were observed in the HFD group. Lysophosphatidylcholines (LPC) total intensities were also found to decrease in the HFD group. However, individual species such as LPC 18:0 and LPC 20:4 were upregulated in the HFD group when they were compared to ND mice. Total lysophosphatidylethanolamines (LPE) were, in addition, downregulated in the HFD group.

#### 2.3.2. Fatty Acids Dysregulation in Hepatic Tissue

Fatty acids constitute the fundamental structural components of complex lipids and can enter the system through dietary intake, be released from visceral adipose tissue during lipolysis, or be synthesized within the liver through DNL. Fourteen (14) liver fatty acids were found statistically significant between the three binary comparisons, as presented in Table 1. More specifically, FA 16:0, FA 18:2, FA 18:3, FA 18:4, FA 20:5, FA 22:5, FA 22:6 were decreased in HFD mice compared to ND, WL, and WLex mice, while FA 16:1, FA 18:1, FA 20:1, FA 20:2, FA 20:3, FA 22:3, FA 22:4 exhibited an increase in the HFD group compared to all other groups. Considering the consistent association between hepatic steatosis and saturated, monounsaturated, and polyunsaturated fatty acids, an investigation of their levels across the studied groups was performed.

The combined levels of saturated fatty acids (SFA, FA 16:0, FA 18:0) in the WL group were found to be similar or even slightly higher in WLex mice compared to ND, whereas in HFD, they were significantly suppressed. The latter has been reported in previous studies of NAFLD animal models [21]. Similar levels are also observed in polyunsaturated fatty acids (PUFA, FA 18:2, FA 18:3, FA 18:4, FA 20:5, FA 22:5, FA 22:6, FA 20:2, FA 20:3, FA 22:3, FA 22:4) of ND, WL, and WLex, whereas their levels in HFD are decreased. However, a reverse pattern was observed in monounsaturated fatty acids (MUFA, FA 16:1, FA 18:1, FA 20:1), which were found to increase in HFD mice compared to all other groups. With regards to non-essential fatty acids (NEFA, FA 16:0, FA 16:1, FA 18:0, FA 18:1), an increase was observed in their levels in HFD groups in comparison to the ND mice and remained at higher levels even after weight loss and exercise. Contrastingly, essential fatty acids (EFA, FA 18:2, FA 18:3) exhibited a suppression in the HFD group, with a trend towards levels closer to those of the ND after weight loss and exercise.

**Figure 4:**
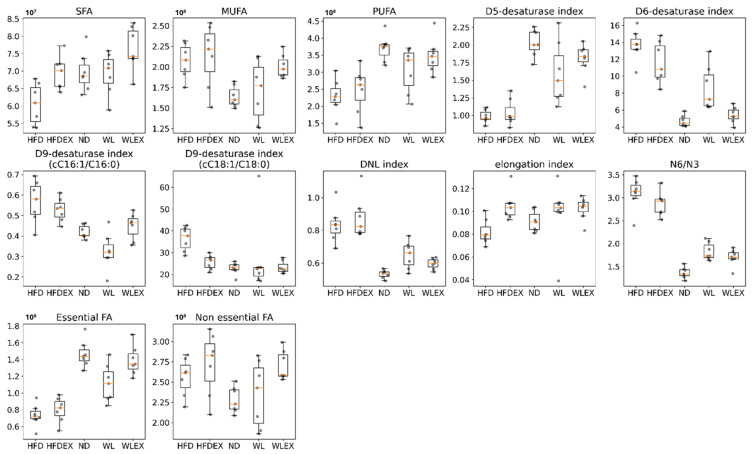
Boxplots showing the distribution of the SFA, MUFA, PUFA, NEFA, EFA, Δ5-desaturase, Δ6-desaturase, Δ9-desaturase, DNL, and elongation indices studied in the studied groups HFD, HFDex, ND, WL, WLex. *p*-values can be found in Table S4.

The lipogenic index derived from the ratio of palmitic acid (FA 16:0) to the essential ω-6 linoleic acid (FA 18:2), which reflects rates of DNL in HFD animals, has shown a reversing trend in WL and WLex. A similar trend was also observed for the desaturation indices Δ6-desaturase (FA 18:2/FA 18:3) and Δ9-desaturase (FA 16:1/FA 16:0 or FA 18:1/FA 18:0), which showed a higher activity of the enzymes in HFD. Interestingly, Δ5-desaturase (FA 18:2/FA 20:4) activity seems to show a decrease in HFD. Nevertheless, the impact of weight loss or exercise seems to reverse this trend, approaching ND levels. Similar findings were observed for the elongation index (FA 18:0/FA 16:0) and the ratio of ω6/ω3 fatty acids. These findings are shown graphically in the box plots in Figure 4. In Table S4, *p*-values and Log2FC values are summarized for all those parameters.

#### 2.3.3. Glycerolipid and Sphingomyelins Dysregulation

Glycerolipids, particularly TGs, are closely linked to the transition from NAFL to NASH. This connection is partially attributed to changes in liver DNL, the rate of lipolysis, and VLDL metabolism. Four (4) TGs with bulk numbers TG(52:2), TG(52:4), TG(56:8), and TG(56:9) were found to be statistically significant between the three binary comparisons. Only TG(52:2) was elevated in the HFD group, a fact that is probably attributed to the higher monounsaturated fatty content (TG 16:1_18:0_18:1). Eight (8) diglycerides were identified as statistically significant among the four groups that were studied, namely DG(36:2), DG(36:3), DG(36:4), DG(38:2), DG(38:3), DG(38:4), DG(40:7), DG(40:8). Only three out of eight DG species, DG(36:3), DG(36:4) and DG(40:8) exhibited lower levels in HFD mice, while all others were found to increase in the HFD group, as demonstrated in Table 1. This trend seems to get reversed with the levels upturning back closer to the ND with exercise and diet. This finding is likely linked to the presence of polyunsaturated fatty acids, particularly FA 18:2 and FA 22:6, in the structure of these three DGs. Concerning SMs, all of them were found to be downregulated in the HFD group compared to ND, WL, and WLex mice.

### 2.4. Visceral Adipose Tissue Triglyceride Profile

**Figure 5:**
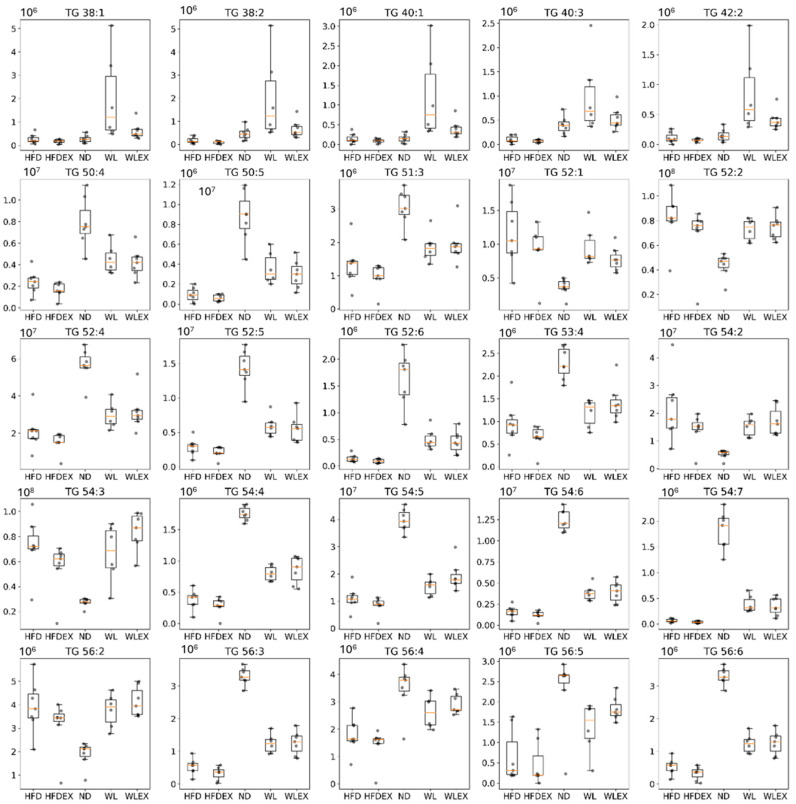
Box plots showing the distribution of TG intensities in the five groups HFD, HFDex, ND, WL, and WLex examined in the study. *p*-values can be found in Table 2.

Insulin resistance in visceral adipose tissue is a key factor leading to increased lipolysis and the release of non-esterified fatty acids into the bloodstream, which is considered the primary metabolic dysfunction in individuals with NAFLD [22]. In this study, the analysis of visceral adipose tissue by OPLS-DA revealed notable differences between HFD mice and those with normal diets, as well as between mice induced to a normal diet for 8 weeks with or without exercise. Similarly, in hepatic tissue, no distinct discrimination was observed between HFD and HFDex mice, nor between the WL and WLex groups, indicating the absence of an impact of the exercise protocol applied on alternating lipid levels in adipose tissue. The validation parameters of the OPLS-DA models can be found in Table S2. Variations among the studied groups were specifically detected in TGs, as they constitute the predominant lipid class in adipose tissue. In the comparison between the HFD and ND, 20 TGs exhibited alterations. When comparing HFD to WL, 5 TGs were altered, and regarding HFD to WLex discrimination, 2 TGs showed changes. Interestingly, the majority of identified TGs exhibited lower concentration levels in HFD compared to ND, WL, and WLex mice. However, an increase in the levels of molecular species of TGs containing saturated fatty acids or/and fatty acids with a low number of double bonds was observed in HFD mice. Specifically, saturated fatty acids such as FA 16:0, FA 18:0, and FA 20:0 and monounsaturated fatty acids, including FA 16:1, FA 18:1, and FA 20:1, were the main fatty acids composed of TGs that were elevated in the HFD group. Additionally, TGs with a low number of carbons (TG 38:1, TG 38:2, TG 40:1, TG 40:3, TG 42:2) were found elevated in the weight loss group. The detailed tables with TG isomer annotations for each comparison are provided in Table S5. Table 2 summarizes all identified lipids that were found significant between HFD-ND, HFD-WL, and HFD-WLex comparisons in visceral adipose tissue along with their statistical parameters, *p*-values, Log2FC, VIP, and CV%, while in Figure 5, significant TGs are illustrated for the different groups in box plots.

## 3. Discussion

In this study, an animal model of NAFLD induced by an HF diet and 5% HFCS was studied based on its hepatic and visceral adipose tissue lipidomic phenotype. The primary focus was directed towards investigating the initial two hallmarks of disease management: the transition to a normal diet and the incorporation of exercise, specifically examining their effects on hepatic and visceral adipose tissue for the first time. Clinical parameters, such as weight loss and histological findings, including steatosis, were examined in conjunction with lipidomic analysis results. The integration of these assessments allowed for a comprehensive conclusion regarding the effectiveness of the animal model in elucidating the mechanisms underlying steatosis.

The analysis of animal body weight records showed significant changes primarily in response to dietary interventions, with exercise playing a secondary role. Both the WL and WLex groups exhibited similar results in terms of weight loss, with a noticeable difference in body weight emerging after the third week of the dietary intervention. The HFDex group demonstrated lower weight loss compared to the dietary intervention groups, suggesting that the influence of diet has a higher impact on body weight in comparison to exercise alone.

When considering the results from the untargeted lipidomic analysis, it also becomes evident that the impact of dietary modification is more influential in shaping hepatic lipid profiles than exercise. This aligns well with our expectations based on the typical NAFLD pathophysiology. As illustrated in the PCA score plots, samples are grouped based on dietary constitution independently of exercise, with samples from the ND group lying close to the WL and WLex groups, suggesting that an 8-week dietary modification, even after 12 weeks of a high-fat diet, could restore mice’s hepatic tissue lipid metabolism to the basal level. When pairwise comparisons between the HFD and HFDex groups, as well as the WL and WLex groups, were considered, discernible discriminations were not identified. Despite the well-established evidence supporting the beneficial effects of exercise on NAFLD, these effects were not clearly apparent on the basis of the lipidome in the studied model.

It is important to note that in a prior study employing an animal model featuring Apolipoprotein E knockout mice exposed to an HFD [23], the authors did not observe a discernible impact of exercise intervention on various biochemical parameters, apart from hepatic transaminases. However, a notable reduction in lipid species, including TG 56:8, TG 52:4, TG 52:2, and epoxy-eicosadiene, was observed when they compared the exercise group to the matched HFD group. In our study, we observed a reduction in TG 56:8 and TG 52:4, along with higher levels of TG 52:2 as well. This alignment with our results suggests a consistent impact of exercise on specific lipid species. One plausible explanation for the observed variations may be the exercise protocol’s duration and frequency, as in the mentioned study [23], which spanned over 12 weeks with 30 to 40 min swimming sessions conducted five days per week [24,25,26]. A very recent comprehensive review [27], encompassing 43 animal studies and 14 randomized clinical trials, systematically investigates the impact of physical activity protocols on the management of NAFLD. The review underscores the significance of standardized exercise durations, highlighting that in animal studies focused on NAFLD management, the exercise intervention ranged from 8 to 12 weeks.

PCs and PEs constitute the predominant phospholipids in mammalian cell membranes. Although no significant variations were observed in PC levels, a notable reduction in PEs was noted. Their relevance in NAFLD is acknowledged, particularly the significance of their ratio, PC/PE. This specific ratio holds crucial importance in various tissues, with both low and high PC/PE ratios correlating with elevated NAS scores in the liver [28]. The balance between PCs and PEs on lipid droplet surfaces is crucial for their dynamics. Inhibiting PC biosynthesis during triglyceride storage conditions enlarges droplets due to altered surface area-to-volume ratios. Additionally, an increased PE presence promotes the fusion of smaller droplets. Adding PC to expanding lipid droplets reduces PE abundance, preventing droplet coalescence [28]. Moreover, diminished concentrations of PCs abundant in PUFAs, specifically those containing FA 22:6, are also evident in NAFLD. The observed decline in the conversion of PE to PC likely signifies a reduction in production via the methionine cycle, impacting the synthesis of S-adenosylmethionine (SAM), which is essential for the enzymatic conversion of PE to PC [29].

The decrease in PI species indicates a connection between disrupted lipid metabolism, inflammation, and hepatic steatosis in NAFLD. In a previous study by Ščupáková [30], the importance of PI and arachidonic acid metabolism in non-steatotic tissue areas was highlighted while linking LDL and VLDL metabolism specifically to steatotic tissue.

Regarding lysophospholipids, our results show significant changes in both LPC and LPE lipid species. Interestingly, changes in LPC in the context of NAFLD/NASH might be related to LPC acyltransferase activity. In the study of Béland-Bonenfant et al., the circulating lipid profiles of 679 patients were examined, analyzing over 400 lipid species to predict hepatic fat content and NAFLD, concluding to lower LPC levels, especially C16:0 and C18:0 for NAFLD patients [29,31]. This observation aligns with our study, where we found significantly lower hepatic levels of LPC 18:0, highlighting the potential relevance of this LPC as a biomarker for NAFLD.

Elevated liver fat content in the TGs and PLs of NAFLD patients is linked to increased levels of TGs containing saturated or monounsaturated fatty acids and reduced levels of phospholipids containing PUFAs [31,32,33,34]. Interestingly, our observations revealed a slight decrease in SFAs, likely attributed to only two identified lipids within this class. We observed an increase in MUFAs and a reduction in PUFAs. The rise in palmitoleic acid (FA 16:1n7), coupled with lower stearic acid (FA 18:0), suggests enhanced Δ9 stearoyl-CoA desaturase activity, consistent with our findings. The increase in palmitoleic acid might represent an adaptive anti-inflammatory reaction to counteract the pro-inflammatory effects of fatty acid overload [29]. Increased dietary intake of ω-6 polyunsaturated fatty acids has raised the ω-6 to ω-3 ratio, contributing to NAFLD. Animal studies support a beneficial reduction in this ratio to address steatosis. Both ω-6 and ω-3 fatty acids undergo oxidation, potentially generating oxylipins through enzymatic or non-enzymatic pathways [32]. Excessive ω-6 may lead to mitochondrial dysfunction, causing cell death. In our model, the estimated ω-6/ω-3 ratio likely increased, aligning with findings in NASH patients by Puri et al. [33].

In exploring additional indicators to unravel the complexities of disrupted metabolism in NAFLD, our investigation unveiled an elevated Δ6 desaturated index and a concurrent reduction in the Δ5 desaturated index associated with the HFD. Significantly, the liver’s desaturation of PUFAs is orchestrated by key enzymes, Δ-6D and Δ-5D, critical for synthesizing essential highly unsaturated FAs like 20:4, n-6, and 22:6, n-3 [35]. Crucially, our findings point to a decline in these specific FAs, mirroring the observed decrease in Δ-6D activity in our study. Importantly, emerging evidence consistently indicates a marked reduction in the activities of both Δ-6D and Δ-5D within the livers of obese NAFLD patients compared to their non-NAFLD counterparts [36]. These insights highlight potential metabolic shifts in NAFLD and underscore the central roles these enzymatic pathways play in the liver’s PUFA desaturation mechanism [37].

In our examination of adipose tissue, the focus shifted to TG content. Utilizing multivariate statistical analysis on profiling data, we observed a pronounced impact of the HFD and, to a lesser extent, exercise on liver outcomes. Prolonged overnutrition and obesity induce a state of reduced metabolic flexibility in adipose tissue, impairing the efficient storage and mobilization of lipids. This metabolic inflexibility, exacerbated by a persistent surplus of circulating free fatty acids (FFAs), extends its systemic effects, influencing the liver and muscle and ultimately contributing to insulin resistance (IR) [38].

Notably, adipose tissue insulin resistance emerges as a crucial factor in driving hepatic fat accumulation and the progression of NAFLD. Insulin’s principal role in adipose tissue involves suppressing lipolysis and facilitating the uptake of fatty acids. This resistance disrupts the regulatory actions of lipases, including adipose tissue triglyceride lipase (ATGL) and hormone-sensitive lipase (HSL), resulting in the release of diacylglycerol and free fatty acids [39,40]. Our findings specifically identified elevated levels of TGs composed of certain fatty acids, such as FA 16:0, FA 18:0, and FA 20:0, alongside MUFAs like FA 16:1, FA 18:1, and FA 20:1 under the HFD. SFAs, particularly 16:0, are recognized as lipotoxic lipids, significantly contributing to chronic inflammation and organ damage in both the liver and adipose tissue [40,41].

We have summarized clinical and microscopic findings, highlighting lipids’ abnormalities, such as shifts in free fatty acid distribution, fluctuations in glycerolipids and sphingolipids, and a diminished PC/PE ratio associated with NAFLD. These alterations likely contribute to the mechanisms driving excessive hepatic triglyceride accumulation, linked to an increased supply of free fatty acids from peripheral adipose tissue to the liver and enhanced de novo lipid synthesis via the lipogenic pathway. This is speculated to potentially result in less liver disposal through *β*-oxidation and VLDL export. Irrespective of the NAFLD stage, the organism endeavors to maintain the compositional integrity of the hepatocyte membrane lipidome, and thus, triglyceride synthesis may be an adaptive, beneficial response when hepatocytes are exposed to potentially toxic triglyceride metabolites. Therefore, advancing our understanding is crucial, not only in tissue-specific mechanisms but also in the cross-talk between different tissues and its systemic impact on the regulation of lipid metabolism [42], considering the vast structural complexity of approximately 40,000 distinct lipids identified to date [43]. Nevertheless, the investigation was limited to specific variations such as the type of high-fat diet and exercise as well as training durations in animal models. These differences in the experimental design of the various studies could influence the pathogenesis and mitigation of NAFLD and consequently be reflected in the related findings [24,27].

## 4. Materials and Methods

### 4.1. Chemicals and Materials

Methanol (MeOH), acetonitrile (ACN), methyl-tert-butyl-ether (MTBE; ≥99%), chloroform (CHCl_3_), and formic acid (all ULC/MS-CC/SFC grade) were obtained from CHEM-LAB NV (Zedelgem, Belgium). Isopropanol (IPA) was purchased from Fisher Scientific (International Inc., Hampton, NH, USA). Ammonium formate (NH_4_HCO_2_; MS grade) was obtained from Sigma-Aldrich (Merck, Darmstadt, Germany). Deionized water (ddH_2_O) was ultrapurified by a Millipore (Bedford, MA, USA) instrument delivering water quality with a resistivity of ≥18.2 MΩ∙cm.

### 4.2. Animal Study

**Figure 6:**
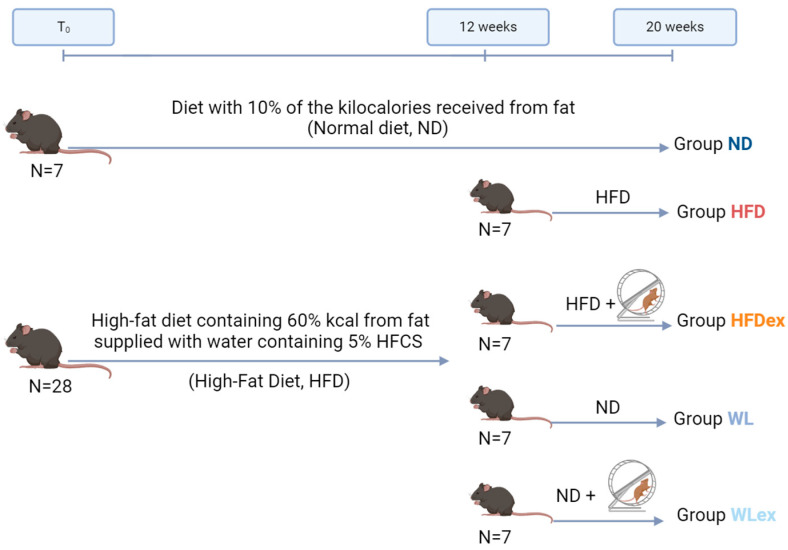
Graphical illustration of the NAFLD mouse model induced by a high-fat diet in combination with 5% HCFS. In the 12 weeks of the experiment, the HFD group was divided into four subgroups: HFD, HFDex, WL, and WLex.

For this study, male mice of the C57BL/6 strain, obtained from the Hellenic Pasteur Institute, were utilized. These animals were housed within the EZEFIS animal facility (Department of Pharmacology, Medical School, NKUA), where the environmental conditions were strictly controlled to maintain a temperature of 20–22 °C, continuous air renewal, and a 12 h light-dark cycle. The mice were provided with unrestricted access to both food and water supplies (ad libitum). Initially, the animal model included two groups. The first group (n = 7) was provided with a normal diet composed 10% kcal from fat (D12450B Research Diets, New Brunswick, NJ, USA) and had access to tap water for a period of 12 weeks. This diet was referred to as a normal diet (ND). The second group (n = 28) consumed a high-fat diet containing 60% kcal from fat (D12492 Research Diets, New Brunswick, NJ, USA) and was supplied with water containing 5% HFCS (Best Flavors, Orange, CA, USA) for 12 weeks. The combination of the high-fat diet and the addition of 5% HFCS to the water was classified as a high-fat diet (HFD). After the end of 12 weeks, the mice previously subjected to HFD were divided randomly into four subgroups: Group a, mice that continued on the same HFD (n = 7); Group b, mice that continued to consume the same HFD and underwent weekly sessions of supervised aerobic exercise (HFD exercise, HFDex, n = 7); Group c, mice that changed diet to ND without the addition of aerobic exercise (Weight Loss, WL, n = 7); and Group d, mice that switched diet to ND and simultaneously placed in weekly sessions of supervised aerobic exercise (Weight Loss exercise, WLex, n = 7). The four subgroups, along with the original ND group, were maintained on their respective diets for an additional eight-week period, and they continued to consume food as previously described. The mice were weighed to monitor their progress every week. In Figure 6, a schematic illustration of the experimental design, including the respective timelines, is provided. The HFDex and WLex feeding groups underwent intense aerobic exercise training, consisting of three 30 min running exercise sessions on a weekly basis, on a treadmill specialized for studies pertinent to exercise and metabolism (PanLab LE8700 Treadmill). Animals were acclimatized to trial exercise sessions on the treadmill. For experiments, mice were urged to achieve a running speed of 20 cm/s. Once the feeding period was complete, the animals were euthanized upon systemic perfusion with phosphate-buffered saline (PBS). Blood, as well as liver and visceral adipose tissues (VAT), were isolated. Animal experiments performed for this study were in accordance with the regulations of the European Union, and the protocol was approved by the Region of Attica, Greece.

### 4.3. Lipidomic Analysis

#### 4.3.1. Liver and Visceral Adipose Tissue Extraction

Liver tissues were transferred to 2.0 mL Eppendorf tubes containing 1.0 mm ceramic beads. An organic solvent mixture of MTBE-MeOH 3:1 (*v*/*v*) was added to the weighed tissue proportionally to weight and up to 1200 μL for the maximum weight. The exact extraction solvent’s volumes are provided in Table S6. Homogenization, followed by performing 4 cycles with a 30 s duration and speed set at 6.00 m/s using a Bead mill Homogenizer (BEAD RUPTOR ELITE, Omni International, Kennesaw, Georgia). The mixture was then centrifuged at 4 °C for 30 min at 10,000 rpm. One hundred and fifty (150) µL of the supernatant were transferred to a 1.5 mL Eppendorf tube and evaporated to dryness in vacuo (SpeedVac, Eppendorf Austria GmbH, Wien, Austria), followed by reconstitution with 150 μL of H_2_O-ACN-IPA in a 1:1:3 (*v*/*v*) ratio.

For visceral adipose tissue lipid extraction, a modified Folch protocol was used. The weighted tissue was transferred to a 2.0 mL Eppendorf tube containing 1.0 mm ceramic beads. The volumes of the extraction solvents were adjusted proportionally to the weighted tissue, as described in Table S6. In 10 mg of adipose tissue, 200 µL of MeOH were added. Homogenization was followed by four cycles of 30 s at 6.00 m/s speed, and the homogenates were transferred to new 2.0 mL Eppendorf tubes, where 640 µL of CHCl_3_-MeOH 7:1 (*v*/*v*) were added. After 30 min of vortexing at room temperature, 360 μL of CHCl_3_ were added and vortexed for another 30 min. One hundred eighty (180) μL of H_2_O were added to enhance phase separation, and the samples were centrifuged for 30 min at 10,000 rpm at 4 °C. Two hundred (200) µL of the lower phase were collected in a new 1.5 mL Eppendorf tube. The extraction process was repeated, and the organic phases were combined and evaporated to dryness, as described above. The dry residues were reconstituted in 200 μL of IPA and diluted 40 times with the same solvent.

For the quality control of the analyses, a pooled sample (Quality Control Sample, QC) was prepared by mixing equal volumes of each supernatant. Phenotypic QC samples were prepared for each of the five groups by mixing equal volumes of the supernatants from the samples of the same group. Diluted QCs (1:2, 1:4, 1:6, 1:8) were also prepared for the evaluation of the dilution integrity of the detected features.

#### 4.3.2. Analytical Instrumentation and Conditions

A UHPLC Elute system equipped with an Elute autosampler operating at 8 °C was used. The separation was performed with an Acquity UPLC CSH C18, 2.1 × 100 mm, 1.7 μm column (Waters Ltd., Elstree, UK) equipped with a pre-column Acquity UPLC CSH C18Van-Guard (Waters Ltd., Elstree, UK), maintained at 55 °C. A 20 min gradient mobile phase system was employed using an HPLC binary solvent manager. The mobile phase A consisted of ACN/H_2_O (60:40), 10 mM ammonium formate, and 0.1% formic acid, and the mobile phase B consisted of IPA/ACN (90:10) and 0.1% formic acid. The gradient was as follows: 60–57% A (0.0–2.0 min), 57–50% A (2.0–2.1 min; curve 1), 50–46% A (2.1–12.0 min), 46–30% A (12.0–12.1 min; curve 1), 30–1% A (12.1–18 min), 1–60% A (18.0–18.1 min), and 60% A (18.1–20.0 min). The flow rate was set at 0.4 mL/min. The injection volume was set at 5 μL in positive mode and at 10 μL in negative mode. The needle was initially washed with the strong wash solvent IPA/ACN (90:10) (1000 μL), followed by the weak wash solvent ACN/H_2_O (60/40) (1000 μL) before and after each injection.

The MS data were acquired using a TIMS TOF mass spectrometer (Bruker, Billerica, MA, USA) in positive and negative ionization modes in liver tissue and only in positive mode for adipose tissue, performing data-dependent acquisition (DDA) for MS/MS analyses. The settings in ESI were as follows: capillary ±4.2 kV, dry temperature 200 °C, dry gas 10 L/min, and nebulizer gas 2 Bar. Auto MS/MS was applied using dynamic MS/MS spectra acquisition with 6 and 10 Hz as minimum and maximum spectra rates, respectively. Collision energy was set at 20 V for precursor ions below 100 *m*/*z*, 30 V for precursor ions with *m*/*z* ranging from 100 to 1000, and 40 V for precursor ions with m/z ranging from 1000 to 2000 *m*/*z*. Calibrant (sodium formate, 10 mM) was infused into MS at a 10 µL/h flow rate in the first 0.2 min of each analysis.

#### 4.3.3. Data Analysis

Raw data from TIMS-TOF were recalibrated using sodium formate clusters by data analysis (version 5.3, Bruker, Bremen, Germany) and converted to mzML by MSConvert (ProteoWizard 3.0.11567). Retention time alignment and feature grouping are performed by XCMS (version 3.2.0) in R programming prior to chromatographic peak detection. Variables containing empty/zero/missing values >50% in each sample group and those with QC CV values >30% were removed. Raw data were normalized using QC samples [44]. SIMCA 13.0.3 (UMETRICS AB Sweden) software was used for unsupervised principal component analysis (PCA), and the data were further processed by partial and orthogonal-partial least squares discriminant analysis (PLS, OPLS-DA). “S-plot” was used, applying absolute p and p(corr) cutoff values of >|0.05| and |0.5|, respectively, for identifying significant features. Parameters that demonstrate the quality of the models, including goodness of fit in the X (R2X) and Y (R2Y) variables and predictability (Q2YCV), were determined by permutation and CV ANOVA analysis. Univariate statistical analysis was performed in the Python programming language to assess the differences between the study groups. The Kruskal–Wallis test was performed, followed by post hoc Bonferroni’s test for multiple comparisons, and statistical significance was defined as a value of *p* ≤ 0.05. The analysis of body weights was conducted using GraphPad Prism v8.0.1 software.

#### 4.3.4. Lipids’ Annotation

Identification of the statistically significant lipid species was performed in Lipostar2 (version 2.0.2, Molecular Discovery Ltd., Hertfordshire, UK) equipped with the LIPID MAPS structure database (version September 2021) [45]. The raw files were imported directly and aligned using the default settings. Automatic peak picking was performed with the Savitzky–Golay algorithm using the following parameters: window size set to 7, degree to 2, multi-pass iterations to 1, and minimum S/N ratio of 3. Mass tolerance settings were set to 10 ppm with an RT tolerance of 0.2 min. Filters “Retain lipids with isotopic pattern” and “Retain lipids with MS/MS” were applied to keep only features with isotopic patterns and MS/MS spectra for identification. The following parameters were used for lipid identification: 5 ppm precursor ion mass tolerance and 20 ppm product ion mass tolerance. The automatic approval was performed to keep structures with a quality of 3–4 stars.

## 5. Conclusions

In the present study, an untargeted lipidomic analysis was used to investigate the effect of weight loss, exercise, and their combination on NAFLD, both in the liver and visceral adipose tissue, for the first time. The results showed disturbed lipid metabolism and variations in important classes such as glycerolipids, glycerophospholipids, and sphingolipids in the HFD animals, which seem to alleviate mainly upon dietary intervention. This finding was not observed with the effects of physical exercise alone. Thus, nutritional intervention has been proven based on the observed lipidomic phenotype as the strongest differentiating factor between the groups, which is in agreement with the histological findings where a remission of NAFLD is observed in these cases.

## Figures and Tables

**Table 1:** Hepatic tissue lipids were found to be significant in the binary group comparisons between HFD-ND, HFD-WL, and HFD-WLEX. *p*-values, Log2FCs, CV% values, and VIP scores are provided for each lipid after univariate Kruskal–Wallis, followed by a post hoc Bonferroni’s test and multivariate analysis. Values highlighted in red correspond to *p* ≤ 0.05 (bold *p* < 0.01) or |Log2FC| ≥1.5. Lipids highlighted in green were found in negative ionization mode.

	HFD-ND			HFD-WL			HFD-WLEX			
Lipids	*p*-Value	Log2FC	VIP	*p*-Value	Log2FC	VIP	*p*-Value	Log2FC	VIP	CV%
DG 36:2	**8.03 × 10** ** ^−^ ** **^5^**	**−3.61**	4.2	**9.75 × 10** ** ^−^ ** **^3^**	−1.43	4.1	2.44 × 10^−^^1^	−0.96	3.4	1.53
DG 36:3	2.55 × 10 ^−^ ^2^	0.27	6.8	1.00 × 10^0^	−0.04	3.6	7.42 × 10^−^^2^	0.24	7.2	0.89
DG 36:4	**3.78 × 10** ** ^−^ ** **^5^**	**2.13**	11	1.00 × 10^0^	0.94	5.9	3.81 × 10 ^−^ ^2^	**1.56**	9.9	1.03
DG 38:2	**3.24 × 10** ** ^−^ ** **^5^**	**−1.81**	4.8	3.12 × 10 ^−^ ^2^	−1.31	5.2	1.63 × 10^−^^1^	−1.17	4.7	1.79
DG 38:3	3.45 × 10 ^−^ ^2^	−0.96	4.2	**3.24 × 10** ** ^−^ ** **^5^**	**−1.58**	5.9	1.50 × 10^−^^1^	−0.88	4.5	6.21
DG 38:4	**5.07 × 10** ** ^−^ ** **^4^**	**−1.66**	4.3	**3.44 × 10** ** ^−^ ** **^3^**	**−1.58**	5.1	1.77 × 10^−^^1^	−0.98	4.1	2.06
DG 40:7	**1.51 × 10** ** ^−^ ** **^2^**	−0.51	5.3	1.00 × 10^0^	−0.11	2.9	7.61 × 10^−^^1^	−0.29	8.4	18.7
DG 40:8	**2.37 × 10** ** ^−^ ** **^5^**	**5.03**	8.0	3.07 × 10^−^^1^	**3.50**	4.5	5.61 × 10^−^^2^	**4.12**	6.2	18.3
**FA 16:0**	1.92 × 10^−^^1^	0.18	4.6	2.64 × 10^−^^1^	0.17	5.4	**2.11 × 10** ** ^−^ ** **^3^**	0.31	7.2	4.68
**FA 16:1**	5.85 × 10^−^^1^	−0.23	3.3	2.55 × 10 ^−^ ^2^	−0.60	6.6	1.00 × 10^0^	−0.02	1.1	3.43
**FA 18:1**	2.55 × 10 ^−^ ^2^	−0.32	9.5	9.17 × 10^−^^1^	−0.18	8.8	1.00 × 10^0^	−0.01	5.4	5.26
**FA 18:2**	**8.64 × 10** ** ^−^ ** **^4^**	0.82	12	2.85 × 10^−^^1^	0.53	11	**4.37 × 10** ** ^−^ ** **^3^**	0.77	14	8.73
**FA 18:3**	**1.08 × 10** ** ^−^ ** **^4^**	**2.38**	8.1	4.76 × 10^−^^1^	1.32	5.7	**2.70 × 10** ** ^−^ ** **^3^**	**2.13**	8.4	3.38
**FA 18:4**	**1.92 × 10** ** ^−^ ** **^4^**	**3.26**	2.5	4.76 × 10^−^^1^	**2.11**	1.8	**1.64 × 10** ** ^−^ ** **^3^**	**3.18**	2.7	3.51
**FA 20:1**	**1.64 × 10** ** ^−^ ** **^3^**	−1.06	4.2	**6.95 × 10** ** ^−^ ** **^3^**	−1.05	5.2	9.72 × 10^−^^2^	−0.81	4.3	12.4
**FA 20:2**	**7.57 × 10** ** ^−^ ** **^4^**	−1.27	3.7	2.30 × 10 ^−^ ^2^	−1.06	4.2	6.16 × 10^−^^2^	−0.94	3.8	2.78
**FA 20:3**	**2.55 × 10** ** ^−^ ** **^4^**	−1.36	3.7	1.38 × 10^−^^1^	−0.85	3.8	1.92 × 10^−^^1^	−0.69	3.3	3.99
**FA 20:5**	**5.96 × 10** ** ^−^ ** **^5^**	**2.95**	11	1.77 × 10^−^^1^	**2.07**	9.1	2.82 × 10 ^−^ ^2^	**2.56**	11	2.58
**FA 22:3**	**3.78 × 10** ** ^−^ ** **^5^**	**−2.86**	2.4	1.16 × 10^−^^1^	**−1.79**	2.7	4.20 × 10 ^−^ ^2^	**−2.08**	2.6	5.01
**FA 22:4**	**9.75 × 10** ** ^−^ ** **^3^**	−1.03	3.7	5.61 × 10^−^^2^	−0.96	4.4	2.30 × 10 ^−^ ^2^	−0.95	4.4	3.68
**FA 22:5**	**3.05 × 10** ** ^−^ ** **^3^**	**1.54**	6.5	5.61 × 10^−^^2^	1.31	6.4	**3.05 × 10** ** ^−^ ** **^3^**	**1.54**	7.6	3.86
**FA 22:6**	**7.57 × 10** ** ^−^ ** **^4^**	0.87	10	6.76 × 10^−^^2^	0.63	10	6.76 × 10^−^^2^	0.65	9.9	2.43
LPC 18:0	1.00 × 10^0^	0.60	2.2	3.12 × 10 ^−^ ^2^	1.07	5.0	1.00 × 10^0^	0.34	2.2	4.23
**LPC 18:2**	8.12 × 10^−^^2^	1.25	5.2	**6.20 × 10** ** ^−^ ** **^3^**	1.41	7.2	**2.39 × 10** ** ^−^ ** **^3^**	1.44	6.5	3.09
**LPC 20:4**	**1.64 × 10** ** ^−^ ** **^3^**	−1.06	4.2	3.12 × 10 ^−^ ^2^	−0.94	4.5	1.00 × 10^0^	−0.44	2.8	4.30
**LPE 18:2**	**6.20 × 10** ** ^−^ ** **^3^**	**2.13**	2.9	2.30 × 10 ^−^ ^2^	**1.85**	3.2	5.61 × 10^−^^2^	**1.77**	2.7	2.68
PC 32:0	**1.64 × 10** ** ^−^ ** **^3^**	0.81	5.5	3.45 × 10 ^−^ ^2^	0.65	5.8	1.50 × 10^−^^1^	0.44	3.3	12.0
PC 32:1	2.85 × 10^−^^1^	0.22	2.5	1.87 × 10 ^−^ ^2^	0.37	4.4	4.63 × 10 ^−^ ^2^	0.29	3.3	4.38
**PC 34:1**	**6.20 × 10** ** ^−^ ** **^3^**	−0.45	3.8	1.00 × 10^0^	−0.01	0.5	6.26 × 10^−^^1^	−0.23	2.9	1.98
**PC 34:2**	**1.26 × 10** ** ^−^ ** **^5^**	0.88	5.5	7.42 × 10^−^^2^	0.64	5.5	1.26 × 10^−^^1^	0.61	4.9	2.66
**PC 34:3**	3.45 × 10 ^−^ ^2^	0.64	2.9	1.00 × 10^0^	−0.04	0.5	1.00 × 10^0^	0.29	1.3	3.60
**PC 36:2**	**1.12 × 10** ** ^−^ ** **^3^**	0.87	4.7	**4.91 × 10** ** ^−^ ** **^3^**	0.85	5.7	4.13 × 10^−^^1^	0.55	3.7	1.63
**PC 36:3**	1.09 × 10 ^−^ ^2^	0.91	3.3	1.00 × 10^0^	0.46	1.6	**8.71 × 10** ** ^−^ ** **^3^**	0.96	3.9	2.63
**PC 36:4**	**2.03 × 10** ** ^−^ ** **^5^**	**1.99**	4.7	3.45 × 10 ^−^ ^2^	1.30	3.8	3.57 × 10^−^^1^	0.98	2.8	4.42
**PC 36:5**	**1.07 × 10** ** ^−^ ** **^5^**	**3.58**	2.7	1.26 × 10^−^^1^	**2.00**	2.0	8.12 × 10^−^^2^	**2.43**	2.0	2.69
PC 38:3	**2.93 × 10** ** ^−^ ** **^4^**	**−1.58**	8.0	1.00 × 10^0^	−0.24	3.1	4.20 × 10 ^−^ ^2^	−0.89	6.9	1.50
PC 38:5	**5.07 × 10** ** ^−^ ** **^4^**	−0.94	5.5	3.81 × 10 ^−^ ^2^	−0.46	5.4	2.30 × 10 ^−^ ^2^	−0.62	5.3	3.27
PC 40:5	6.76 × 10^−^^2^	1.25	2.8	**8.71 × 10** ** ^−^ ** **^3^**	1.33	3.3	1.35 × 10 ^−^ ^2^	1.36	3.4	3.35
PC 40:6	1.00 × 10^0^	0.07	3.6	**9.75 × 10** ** ^−^ ** **^3^**	0.20	7.1	1.00 × 10^0^	0.09	3.9	2.97
PC 40:8	3.12 × 10 ^−^ ^2^	0.96	5.5	**3.05 × 10** ** ^−^ ** **^3^**	1.09	7.4	1.51 × 10 ^−^ ^2^	0.99	6.3	1.27
**PE 34:2**	**5.80 × 10** ** ^−^ ** **^4^**	**1.57**	5.1	8.89 × 10^−^^2^	1.19	5.4	4.44 × 10^−^^1^	0.95	3.8	1.39
**PE 36:2**	**3.78 × 10** ** ^−^ ** **^5^**	1.34	5.2	1.87 × 10 ^−^ ^2^	1.07	5.4	2.26 × 10^−^^1^	0.94	4.7	1.57
**PE 36:3**	**9.31 × 10** ** ^−^ ** **^5^**	1.35	4.6	3.57 × 10^−^^1^	0.81	3.9	1.26 × 10^−^^1^	0.80	3.1	1.25
PE 36:4	**6.92 × 10** ** ^−^ ** **^5^**	**2.26**	11	9.72 × 10^−^^2^	**2.06**	12	3.45 × 10 ^−^ ^2^	**2.07**	11	0.95
**PE 36:5**	1.21 × 10 ^−^ ^2^	**2.23**	2.8	**2.39 × 10** ** ^−^ ** **^3^**	**2.33**	3.6	2.07 × 10 ^−^ ^2^	**2.16**	3.2	3.64
PE 38:5	6.68 × 10^−^^1^	1.05	2.6	**3.86 × 10** ** ^−^ ** **^4^**	**1.64**	4.9	1.35 × 10 ^−^ ^2^	1.32	3.8	1.08
**PE 38:6**	**3.86 × 10** ** ^−^ ** **^4^**	0.79	5.9	1.77 × 10^−^^1^	0.42	4.8	1.63 × 10^−^^1^	0.42	4.4	1.83
**PE 38:7**	**9.31 × 10** ** ^−^ ** **^5^**	1.22	2.8	3.31 × 10^−^^1^	0.67	2.1	1.38 × 10^−^^1^	0.74	2.1	1.30
**PE 40:6**	3.45 × 10 ^−^ ^2^	0.60	2.9	2.30 × 10 ^−^ ^2^	0.50	3.1	1.16 × 10^−^^1^	0.43	2.5	1.43
**PE O-38:5**	1.00 × 10^0^	−0.36	1.5	1.51 × 10 ^−^ ^2^	−0.93	2.7	1.35 × 10 ^−^ ^2^	−0.83	2.5	2.50
**PI 34:2**	**2.78 × 10** ** ^−^ ** **^5^**	**1.81**	3.5	9.72 × 10^−^^2^	1.30	3.4	6.16 × 10^−^^2^	1.29	3.2	4.91
**PI 38:5**	1.87 × 10 ^−^ ^2^	**2.84**	2.1	**7.57 × 10** ** ^−^ ** **^4^**	**3.08**	2.8	3.45 × 10 ^−^ ^2^	**2.65**	2.2	2.58
**PS 38:6**	**4.43 × 10** ** ^−^ ** **^4^**	**1.70**	3.3	7.13 × 10^−^^1^	0.77	2.0	1.00 × 10^0^	0.35	1.2	2.88
**SM 40:1;O2**	**2.55 × 10** ** ^−^ ** **^4^**	1.48	4.8	5.10 × 10^−^^1^	0.79	3.4	1.00 × 10^0^	0.50	2.5	4.90
SM 41:1;O2	**3.44 × 10** ** ^−^ ** **^3^**	1.20	4.3	**2.70 × 10** ** ^−^ ** **^3^**	1.25	5.2	1.63 × 10^−^^1^	0.98	3.7	1.00
SM 42:1;O2	**2.78 × 10** ** ^−^ ** **^5^**	1.23	6.7	2.85 × 10^−^^1^	0.98	6.6	1.68 × 10 ^−^ ^2^	1.07	6.6	6.28
TG 52:2	**4.43 × 10** ** ^−^ ** **^4^**	−1.35	4.1	2.85 × 10^−^^1^	−0.71	3.6	4.44 × 10^−^^1^	−0.69	2.7	12.6
TG 52:4	**6.20 × 10** ** ^−^ ** **^3^**	0.53	4.3	1.00 × 10^0^	0.23	2.3	2.08 × 10^−^^1^	0.45	3.9	1.65
TG 56:8	2.08 × 10^−^^1^	0.92	3.2	**7.79 × 10** ** ^−^ ** **^3^**	1.12	4.2	6.76 × 10^−^^2^	0.90	3.3	5.10
TG 56:9	**3.24 × 10** ** ^−^ ** **^5^**	**1.63**	3.9	4.13 × 10^−^^1^	1.00	2.6	1.38 × 10^−^^1^	1.24	3.4	3.78

**Table 2:** Visceral adipose tissue lipids were identified as significant in the group comparisons between HFD-ND, HFD-WL, and HFD-WLex. *p*-values, Log2FCs, QC CV values, and VIP scores are provided for each lipid after univariate Kruskal–Wallis, followed by a post hoc Bonferroni’s test and multivariate analysis. Values highlighted in red correspond to *p* ≤ 0.05 or |Log2FC| ≥ 1.5.

	HFD-ND			HFD-WL			HFD-WLex			
Compounds	*p*-Values	Log2FC	VIP	*p*-Values	Log2FC	VIP	*p*-Values	Log2FC	VIP	CV%
TG 38:1	1.00 × 10^0^	−0.01	0.3	**7.44 × 10** ** ^−^ ** **^3^**	**2.87**	2.6	2.60 × 10^−^^1^	1.19	1.0	5.13
TG 38:2	2.82 × 10^−^^1^	**1.52**	0.6	**5.94 × 10** ** ^−^ ** **^4^**	**3.57**	3.0	5.71 × 10^−^^2^	2.00	1.3	4.75
TG 40:1	1.00 × 10^0^	−0.01	0.2	**5.17 × 10** ** ^−^ ** **^3^**	**3.04**	2.8	1.87 × 10^−^^1^	1.42	0.9	5.77
TG 40:3	1.32 × 10^−^^1^	**2.01**	0.7	**6.79 × 10** ** ^−^ ** **^4^**	**3.33**	3.0	1.17 × 10^−^^2^	**2.42**	1.3	3.92
TG 42:2	1.00 × 10^0^	0.43	0.2	**2.74 × 10** ** ^−^ ** **^3^**	**2.93**	2.8	2.27 × 10^−^^2^	**1.89**	1.0	6.83
TG 50:4	**2.40 × 10^−^^4^**	**1.72**	3.1	2.19 × 10^−^^1^	0.91	1.6	4.14 × 10^−^^1^	0.82	2.4	4.12
TG 50:5	**4.51 × 10^−^^5^**	**3.23**	1.2	1.21 × 10^−^^1^	1.95	2.6	2.60 × 10^−^^1^	1.68	0.8	6.50
TG 51:3	**5.63 × 10^−^^4^**	1.20	1.8	1.00 × 10⁰	0.49	1.2	5.94 × 10^−^^1^	0.55	1.4	5.03
TG 52:1	**1.11 × 10^−^^3^**	**−1.65**	3.7	1.00 × 10⁰	−0.26	0.9	9.44 × 10^−^^1^	−0.56	3.6	3.97
TG 52:2	**1.66 × 10^−^^3^**	−0.90	8.1	1.00 × 10⁰	−0.17	0.8	1.00 × 10^0^	−0.14	5.7	2.40
TG 52:4	**3.20 × 10^−^^4^**	1.42	8.0	8.20 × 10^−^^1^	0.49	1.2	7.78 × 10^−^^1^	0.57	5.7	4.88
TG 52:5	**3.29 × 10^−^^5^**	**2.33**	4.6	1.45 × 10^−^^1^	1.07	1.7	3.56 × 10^−^^1^	0.95	2.8	4.21
TG 52:6	**1.14 × 10^−^^4^**	**3.48**	1.6	1.91 × 10^−^^1^	1.78	2.5	3.10 × 10^−^^1^	1.63	1.1	9.23
TG 53:4	**8.51 × 10^−^^4^**	1.28	1.6	1.00 × 10⁰	0.34	1.1	6.36 × 10^−^^1^	0.59	1.2	4.42
TG 54:2	**1.66 × 10^−^^3^**	**−2.04**	5.3	1.00 × 10⁰	−0.50	1.0	1.00 × 10^0^	−0.33	4.3	4.06
TG 54:3	3.12 × 10^−^^2^	−1.42	8.9	1.00 × 10⁰	−0.13	0.7	1.00 × 10^0^	0.21	5.6	2.94
TG 54:4	**2.02 × 10^−^^5^**	**2.22**	1.6	2.43 × 10^−^^1^	1.09	1.7	1.11 × 10^−^^1^	1.19	1.4	3.68
TG 54:5	**1.14 × 10^−^^4^**	**1.84**	7.2	1.00 × 10⁰	0.48	1.1	2.03 × 10^−^^1^	0.80	5.2	3.41
TG 54:6	**2.02 × 10^−^^5^**	**2.98**	4.5	1.84 × 10^−^^1^	1.28	1.8	1.44 × 10^−^^1^	1.32	3.0	4.10
TG 54:7	**7.18 × 10^−^^5^**	**4.84**	1.8	2.09 × 10^−^^1^	2.61	2.9	2.62 × 10^−^^1^	2.44	1.1	10.2
TG 56:2	2.81 × 10^−^^2^	−1.05	1.9	1.00 × 10⁰	−0.06	0.6	1.00 × 10^0^	0.07	1.1	3.19
TG 56:3	**2.38 × 10^−^^5^**	**2.63**	2.2	1.99 × 10^−^^1^	1.22	1.8	1.72 × 10^−^^1^	1.24	1.6	3.54
TG 56:4	**1.66 × 10^−^^3^**	0.95	1.6	6.35 × 10^−^^1^	0.55	1.1	1.21 × 10^−^^1^	0.70	1.9	3.21
TG 56:5	**2.44 × 10^−^^3^**	**1.81**	1.5	8.91 × 10^−^^1^	1.06	1.4	1.72 × 10^−^^1^	1.48	1.9	6.52
TG 56:6	**2.38 × 10^−^^5^**	**2.63**	2.2	1.99 × 10^−^^1^	1.22	1.8	1.72 × 10^−^^1^	1.24	1.6	3.54

## Data Availability

Data are contained within the article and Supplementary Materials.

## Supplementary Materials

The following supporting information can be downloaded at: https://www.mdpi.com/article/10.3390/molecules29071494/s1, Figures S1–S3. PCA score plots of mice hepatic and adipose tissue samples of the five studied groups and QC samples. All groups (HFD, HFDex, ND, WL, WLex) are illustrated with grey color while QC samples are presented with purple color and clustered together. Figure S4. PLS score plot of mice adipose tissue samples of the five studied groups. HFD and HFDex groups are clustered together on the negative part of the y-axis, ND group is in the center of the eclipse, while WL and WLex mice are grouped together on the positive side of the same axis; Table S1: Comparisons of the mice body weights among the groups HFD-HFDex, HFD-WL, HFD-WLex, ND-WL, ND-WLex during diet and exercise innervations. *p* value < 0.05 were indicated as significant and highlighted; Table S2: Characteristics of the constructed unsupervised and supervised models, log transformation and pareto (PAR) scale were used in all models in Liver -ESI and adipose tissue +ESI, while only pareto (PAR) scale was used in Liver +ESI models; Table S3: Summary of all statistically identified lipids in the liver of mice for the three binary comparisons, HFD-ND, HFD-WL, HFD-WLex. Information is provided regarding the structural formula and fatty acid chains of the lipids, molecular structure, monoisotopic mass, detected derivatives, retention time, and mass accuracy; Table S4: P values and Log2FC for all sums of fatty acids and indices studied in the comparisons HFD-ND, HFD-WL, HFD-WLex; Table S5: Summary of all statistically significant identified lipids in adipose tissue of mice for the three binary comparisons, HFD-ND, HFD-WL, HFD-WLex. Information is provided regarding the structural formula and fatty acid chains of the lipids, molecular structure, monoisotopic mass, detected derivatives, retention time, and mass accuracy; Table S6: Description of the extractions performed on the mice liver and adipose tissue samples.
