# Supplementary material for: Lipidomic Analysis of Liver and Adipose Tissue in a High-Fat Diet-Induced Non-Alcoholic Fatty Liver Disease Mice Model Reveals Alterations in Lipid Metabolism by Weight Loss and Aerobic Exercise
Source: Molecules. 2024 Mar 27;29(7):1494. doi: 10.3390/molecules29071494 (PMC11013466; doi:10.3390/molecules29071494)
Supplement: Supplementary file 1 [file molecules-29-01494-s001.zip › Supplementary Figures_final.pdf]

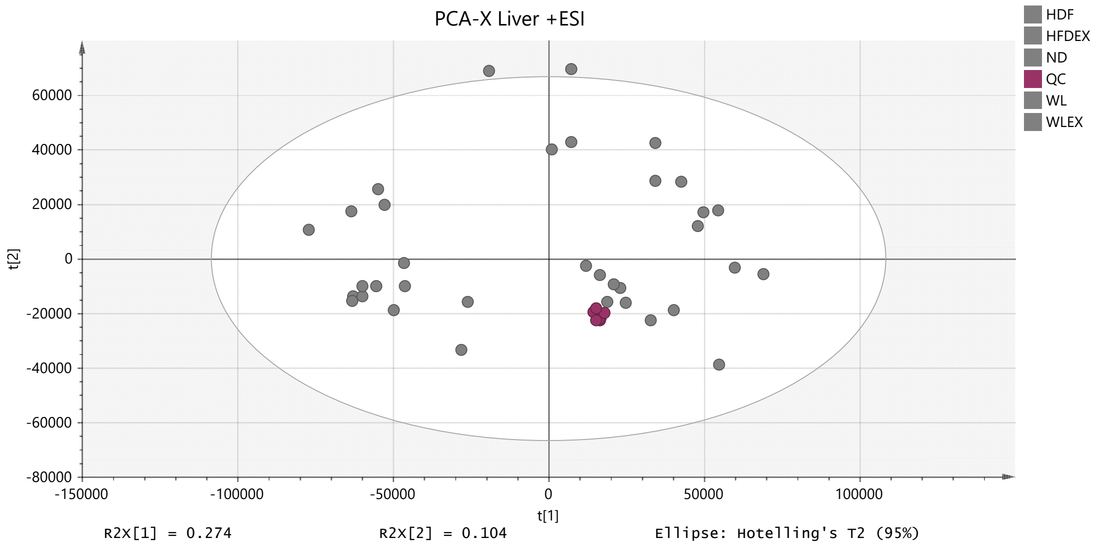
PCA-X Liver +ESI
HDF
HFDEX
ND
QC
WL
WLEX
t[1]
t[2]
R2X[1] = 0.274
R2X[2] = 0.104
Ellipse: Hotelling's T2 (95%)

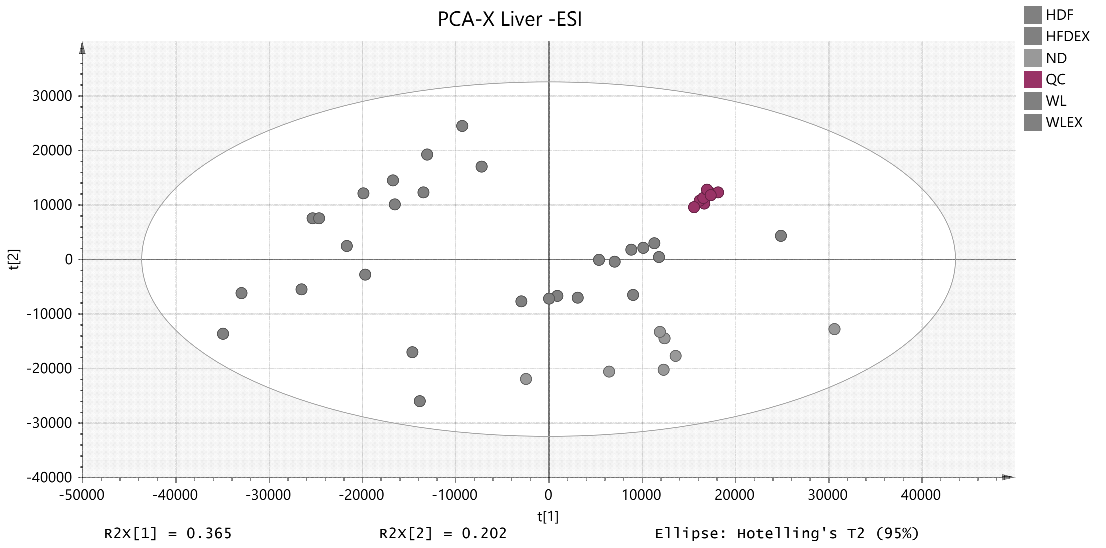
PCA-X Liver -ESI
HDF
HFDEX
ND
QC
WL
WLEX
t[1]
t[2]
R2X[1] = 0.365
R2X[2] = 0.202
Ellipse: Hotelling's T2 (95%)

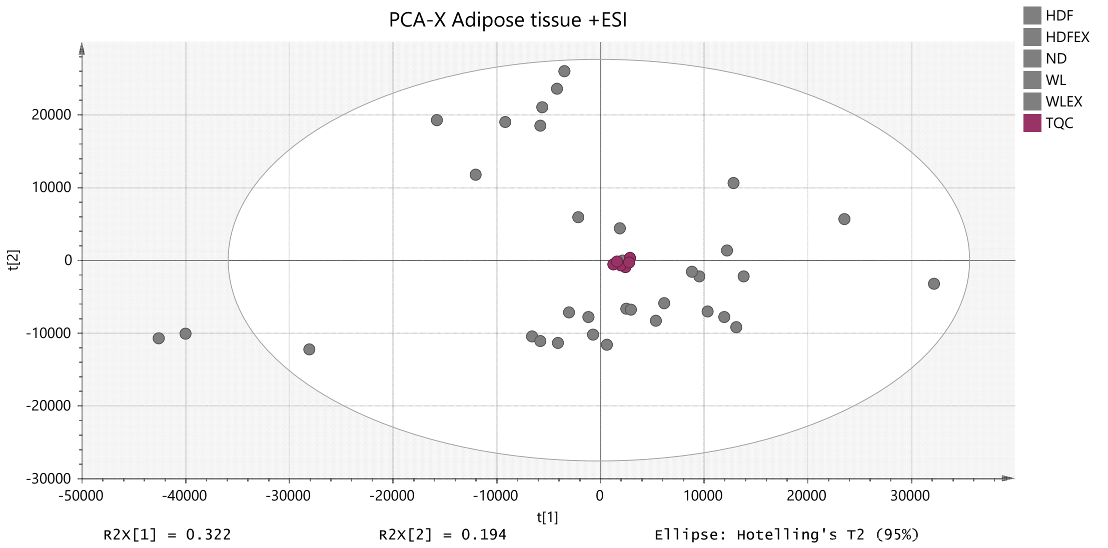
PCA-X Adipose tissue +ESI
HDF
HDFEX
ND
WL
WLEX
TQC
t[1]
t[2]
R2X[1] = 0.322
R2X[2] = 0.194
Ellipse: Hotelling's T2 (95%)

Figure S1-S3. PCA score plots of mice hepatic and adipose tissue samples of the five studied groups and QC samples. All groups (HFD, HFDex, ND, WL, WLex) are illustrated with grey color while QC samples are presented with purple color and clustered together.

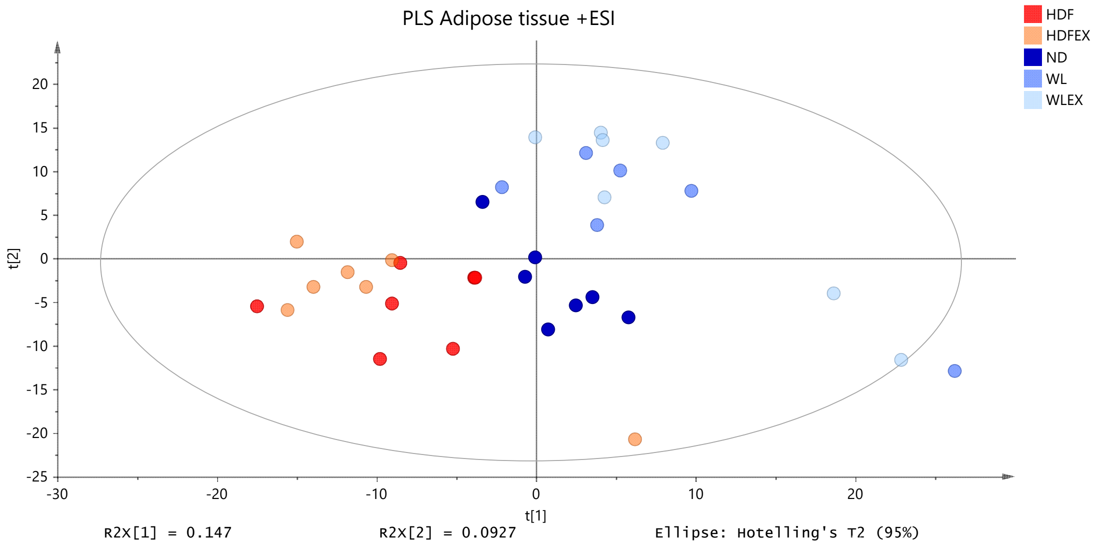

Figure S4 a. PLS score plot of mice adipose tissue samples of the five studied groups. HFD and HFDex groups are clustered together on the negative part of the y-axis, ND group is in the center of the eclipse, while WL and WLex mice are grouped together on the positive side of the same axis.
