# Supplementary material for: Lipidomic Analysis of Liver and Adipose Tissue in a High-Fat Diet-Induced Non-Alcoholic Fatty Liver Disease Mice Model Reveals Alterations in Lipid Metabolism by Weight Loss and Aerobic Exercise
Source: Molecules. 2024 Mar 27;29(7):1494. doi: 10.3390/molecules29071494 (PMC11013466; doi:10.3390/molecules29071494)
Supplement: Supplementary file 1 [file molecules-29-01494-s001.zip › Supplementary Tables_final.pdf]

Supplementary table S1. Comparisons of the mice body weights among the groups HFD-HFDex, HFD-WL, HFD-WLex, ND-WL, ND-WLex during diet and exercise innervations. P values<0.05 were indicated as significant and highlighted.

Week of intervation	week 0	week 1	week 2	week 3	week 4	week 5	week 6	week 7	week 8
Week of the experiment	week 12	week 13	week 14	week 15	week 16	week 17	week 18	week 19	week 20
HFD-HFDex	8.14E-01	6.76E-01	4.19E-01	2.30E-01	1.37E-01	1.51E-01	1.37E-01	1.67E-01	2.01E-01
HFD-WL	7.35E-01	6.11E-01	2.33E-02	4.46E-02	7.20E-03	2.10E-03	3.50E-03	3.00E-04	3.00E-04
HFD-WLex	6.58E-01	6.50E-02	1.70E-03	1.10E-02	6.00E-04	7.00E-04	1.00E-04	5.00E-04	4.00E-04
ND-WL	8.00E-04	1.50E-03	1.32E-02	1.32E-02	8.52E-02	3.75E-01	3.89E-01	8.14E-01	5.49E-01
ND-WLex	1.06E-02	6.40E-02	1.06E-01	2.20E-01	3.22E-01	5.66E-01	9.79E-01	9.38E-01	6.29E-01

Supplementary table S2. Characteristics of the constructed unsupervised and supervised models, log transformation and pareto (PAR) scale were used in all models in Liver -ESI and adipose tissue +ESI, while only pareto (PAR) scale was used in Liver +ESI models.

**Model**	**Type**	**N**	**R2X(cum)**	**R2Y(cum)**	**Q2(cum)**	**CV ANOVA**
Liver +ESI						
ND-HFD-HFDex-WL-WLex_QC	PCA-X	42	0.552		0.348	
ND-HFD-HFDex-WL-WLex	PCA-X	35	0519		0.349	
HFD-ND	OPLS-DA	14	0.507	0.996	0.946	1.04e-005
HFD-WL	OPLS-DA	14	0.404	0.991	0.912	8.92e-005
HFD-WLex	OPLS-DA	14	0.441	0.989	0.914	8.04e-005
Liver -ESI						
ND-HFD-HFDex-WL-WLex_QC	PCA-X	43	0.674		0.539	
ND-HFD-HFDex-WL-WLex	PCA-X	35	0.617		0.467	
HFD-ND	OPLS-DA	14	0.608	0.991	0.955	4.61e-006
HFD-WL	OPLS-DA	14	0.533	0.973	0.915	7.81e-005
HFD-WLex	OPLS-DA	14	0.569	0.985	0.936	2.14e-005
Adipose tissue +ESI						
ND-HFD-HFDex-WL-WLex_QC	PCA-X	42	0.714		0.517	
ND-HFD-HFDex-WL-WLex	PCA-X	34	0.742		0.522	
ND-HFD-HFDex-WL-WLex	PLS	34	0.51	0.312	0.152	6.58e-006
HFD-ND	OPLS-DA	14	0.696	0.983	0.952	6.90e-006
HFD-WL	OPLS-DA	13	0.407	0.928	0.712	3.10e-02
HFD-WLex	OPLS-DA	14	0.555	0.937	0.776	5.00E-03

Supplementary Table S3. Summary of all statistically identified lipids in the liver of mice for the three binary comparisons, HFD_ND, HFD_WL, HFD_WLex. Information is provided regarding the structural formula and fatty acid chains of the lipids, molecular structure, monoisotopic mass, detected derivatives, retention time, and mass accuracy.

HFD-ND									
a/a	Bulk number	Annotations	Neutral Formula	Exact mass m/z	Monoisotopic mass	Monoisotopic mass adduct	D(ppm)	Rt min	Adduct
1	DG 36:2	DG 18:1_18:1	C39H72O5	638.5662	620.5380	638.5718	-8.72	13.6	[M+NH4]+
2	DG 36:3	DG 18:1_18:2	C39H70O5	636.5591	618.5223	636.5561	4.71	11.8	[M+NH4]+
3	DG 36:4	DG 18:2_18:2	C39H68O5	634.5395	616.5067	634.5405	-1.58	9.4	[M+NH4]+
4	DG 38:2	DG 18:1_20:1	C41H76O5	666.5978	648.5692	666.6031	-7.94	13.8	[M+NH4]+
5	DG 38:3	DG 18:2_20:1	C41H74O5	664.5830	646.5536	664.5874	-6.55	13.3	[M+NH4]+
6	DG 38:4	DG 18:1_20:3	C41H72O5	662.5709	644.5379	662.5718	-1.31	12.6	[M+NH4]+
7	DG 40:7	DG 18:1_22:6	C43H70O5	684.5607	666.5223	684.5561	6.68	10.2	[M+NH4]+
8	DG 40:8	DG 18:2_22:6	C43H68O5	682.5357	664.5066	682.5405	-6.97	8.1	[M+NH4]+
9	FA 16:0	FA 16:0	C16H32O2	255.2354	256.2402	255.2330	9.55	2.7	[M-H]-
10	FA 16:1	FA 16:1	C16H30O2	253.2178	254.2246	253.2173	2.05	2.0	[M-H]-
11	FA 18:1	FA 18:1	C18H34O2	281.2483	282.2559	281.2486	-1.07	2.8	[M-H]-
12	FA 18:2	FA 18:2	C18H32O2	279.2335	280.2402	279.2330	1.79	2.2	[M-H]-
13	FA 18:3	FA 18:3	C18H30O2	277.2171	278.2246	277.2173	-0.74	1.7	[M-H]-
14	FA 18:4	FA 18:4	C18H28O2	275.2012	276.2089	275.2017	-1.75	1.4	[M-H]-
15	FA 20:1	FA 20:1	C20H38O2	309.2801	310.2872	309.2799	0.80	3.7	[M-H]-
16	FA 20:2	FA 20:2	C20H36O2	307.2638	308.2715	307.2643	-1.65	3.0	[M-H]-
17	FA 20:3	FA 20:3	C20H34O2	305.2482	306.2559	305.2486	-1.24	2.6	[M-H]-
18	FA 20:5	FA 20:5	C20H30O2	301.2164	302.2246	301.2173	-3.09	1.6	[M-H]-
19	FA 22:3	FA 22:3	C22H38O2	333.2800	334.2872	333.2799	0.39	3.3	[M-H]-
20	FA 22:4	FA 22:4	C22H36O2	331.2642	332.2715	331.2643	-0.43	2.7	[M-H]-
21	FA 22:5	FA 22:5	C22H34O2	329.2486	330.2559	329.2486	0.14	2.1	[M-H]-
22	FA 22:6	FA 22:6	C22H32O2	327.2317	328.2402	327.2330	-4.10	1.9	[M-H]-
23	LPC 18:2	LPC 18:2	C26H50NO7P	564.3304	519.3325	564.3307	-0.57	1.0	[M+HCOO]-
24	LPC 20:4	LPC 20:4	C28H50NO7P	588.3303	543.3325	588.3307	-0.73	1.0	[M+HCOO]-
25	LPE 18:2	LPE 18:2	C23H44NO7P	476.2781	477.2855	476.2783	-0.34	1.1	[M-H]-

26	PC 32:0	PC 16:0_16:0	C40H80NO8P	734.5721	733.5622	734.5694	3.68	8.0	[M+H]+
27	PC 32:1	PC 16:0_16:1	C40H78NO8P	732.5473	731.5465	732.5538	-8.85	6.3	[M+H]+
28	PC 34:1	PC 16:0_18:1	C42H82NO8P	804.5732	759.5778	804.5760	-3.50	8.3	[M+HCOO]-
29	PC 34:2	PC 16:0_18:2	C42H80NO8P	802.5575	757.5622	802.5604	-3.61	6.7	[M+HCOO]-
30	PC 34:3	PC 16:1_18:2	C42H78NO8P	800.5412	755.5465	800.5447	-4.36	5.3	[M+HCOO]-
31	PC 36:2	PC 18:0_18:2	C44H84NO8P	830.5889	785.5935	830.5917	-3.43	8.8	[M+HCOO]-
32	PC 36:3	PC 18:1_18:2	C44H82NO8P	828.5724	783.5778	828.5760	-4.34	6.8	[M+HCOO]-
33	PC 36:4	PC 18:2_18:2	C44H80NO8P	826.5625	781.5622	826.5604	2.52	5.6	[M+HCOO]-
34	PC 36:5	PC 18:2_18:3	C44H78NO8P	824.5419	779.5465	824.5447	-3.34	4.6	[M+HCOO]-
35	PC 36:5	PC 16:0_20:5	C44H78NO8P	780.5514	779.5465	780.5538	-3.09	5.4	[M+H]+
36	PC 38:3	PC 18:0_20:3	C46H86NO8P	812.6161	811.6091	812.6164	-0.37	9.5	[M+H]+
37	PC 38:5	PC 18:1_20:4	C46H82NO8P	808.5868	807.5778	808.5851	2.10	6.6	[M+H]+
38	PC 40:5	PC 18:0_22:5	C48H86NO8P	836.6210	835.6091	836.6164	5.47	8.6	[M+H]+
39	PC 40:8	PC 18:2_22:6	C48H80NO8P	830.5625	829.5621	830.5694	-8.30	5.0	[M+H]+
40	PE 34:2	PE 16:0_18:2	C39H74NO8P	714.5114	715.5152	714.5079	4.86	7.2	[M-H]-
41	PE 36:2	PE 18:0_18:2	C41H78NO8P	742.5405	743.5465	742.5392	1.82	9.6	[M-H]-
42	PE 36:3	PE 18:1_18:2	C41H76NO8P	740.5297	741.5309	740.5236	8.20	7.4	[M-H]-
43	PE 36:4	PE 16:0_20:4	C41H74NO8P	740.5168	739.5152	740.5225	-7.68	7.0	[M+H]+
44	PE 36:5	PE 16:0_20:5	C41H72NO8P	736.4925	737.4996	736.4923	0.30	5.7	[M-H]-
45	PE 38:5	PE 18:0_20:5	C43H76NO8P	766.5408	765.5309	766.5381	3.54	7.6	[M+H]+
46	PE 38:6	PE 16:0_22:6	C43H74NO8P	762.5050	763.5152	762.5079	-3.80	6.5	[M-H]-
47	PE 38:7	PE 16:1_22:6	C43H72NO8P	760.4950	761.4996	760.4923	3.55	5.0	[M-H]-
48	PE 40:6	PE 18:0_22:6	C45H78NO8P	790.5354	791.5465	790.5392	-4.84	8.5	[M-H]-
49	PI 34:2	PI 16:0_18:2	C43H79O13P	833.5206	834.5258	833.5186	2.43	4.8	[M-H]-
50	PI 38:5	PI 18:0_20:5	C47H81O13P	883.5341	884.5415	883.5342	-0.09	5.1	[M-H]-
51	PS 38:6	PS 16:0_22:6	C44H74NO10P	806.4998	807.5050	806.4978	2.47	4.6	[M-H]-
52	SM 40:1;O2	SM 40:1;O2	C45H91N2O6P	831.6553	786.6615	831.6597	-5.29	13.1	[M+HCOO]-
53	SM 41:1;O2	SM 41:1;O2	C46H93N2O6P	801.6883	800.6771	801.6844	4.86	13.4	[M+H]+
54	SM 42:1;O2	SM 42:1;O2	C47H95N2O6P	815.6974	814.6928	815.7000	-3.25	13.7	[M+H]+
55	TG 52:2	TG (16:0_18:0_18:2)<br>TG (16:1_18:0_18:1)	C55H102O6	876.8034	858.7676	876.8015	2.13	16.5	$[M+NH_4]+$

a/a	Bulk number	Annotations	Neutral Formula	Exact mass m/z	Monoisotopic mass	Monoisotopic mass adduct	D(ppm)	Rt min	Adduct
56	TG 52:4	TG (16:0_18:1_18:3)<br>TG(16:0_16:1_20:3)<br>TG (14:0_18:1_20:3)<br>TG (16:0_18:2_18:2)<br>TG (16:1_16:1_20:2)	C55H98O6	872.7750	854.7363	872.7702	5.50	15.5	[M+NH4]+
57	TG 56:8	TG (16:0_18:2_22:6)<br>TG (16:1_18:2_22:5)<br>TG (16:0_20:4_20:4)<br>TG(18:2_18:1_20:5)<br>TG(18:2_18:2_20:4)<br>TG (16:1_20:2_20:5)	C59H98O6	920.7700	902.7363	920.7702	-0.22	15.3	[M+NH4]+
58	TG 56:9	TG (18:2_18:3_20:4)<br>TG (16:1_18:2_22:6)<br>TG (16:1_18:3_22:5)<br>TG (18:2_18:2_20:4)	C59H96O6	918.7516	900.7207	918.7545	-3.20	15.0	[M+NH4]+
HDF_WL									
a/a	Bulk number	Annotations	Neutral Formula	Exact mass m/z	Monoisotopic mass	Monoisotopic mass adduct	D(ppm)	Rt min	Adduct
1	DG 36:2	DG 18:1_18:1	C39H72O5	638.5662	620.5380	638.5718	-8.72	13.6	[M+NH4]+
2	DG 36:4	DG 18:2_18:2	C39H68O5	634.5395	616.5067	634.5405	-1.58	9.4	[M+NH4]+
3	DG 38:2	DG 18:1_20:1	C41H76O5	666.5978	648.5692	666.6031	-7.94	13.8	[M+NH4]+
4	DG 38:3	DG 18:2_20:1	C41H74O5	664.5830	646.5536	664.5874	-6.55	13.3	[M+NH4]+
5	DG 38:4	DG 18:1_20:3	C41H72O5	662.5709	644.5379	662.5718	-1.31	12.6	[M+NH4]+
6	DG 40:7	DG 18:1_22:6	C43H70O5	684.5566	666.5223	684.5561	0.73	10.2	[M+NH4]+
7	DG 40:8	DG 18:2_22:6	C43H68O5	682.5357	664.5066	682.5405	-6.97	8.1	[M+NH4]+
8	FA 16:0	FA 16:0	C16H32O2	255.2338	256.2402	255.2330	3.13	2.7	[M-H]-
9	FA 16:1	FA 16:1	C16H30O2	253.2178	254.2246	253.2173	2.05	2.0	[M-H]-
10	FA 18:2	FA 18:2	C18H32O2	279.2335	280.2402	279.2330	1.79	2.2	[M-H]-
11	FA 18:3	FA 18:3	C18H30O2	277.2171	278.2246	277.2173	-0.74	1.7	[M-H]-
12	FA 18:4	FA 18:4	C18H28O2	275.2012	276.2089	275.2017	-1.75	1.4	[M-H]-
13	FA 20:1	FA 20:1	C20H38O2	309.2801	310.2872	309.2799	0.80	3.7	[M-H]-

14	FA 20:2	FA 20:2	C20H36O2	307.2638	308.2715	307.2643	-1.65	3.0	[M-H]-
15	FA 20:3	FA 20:3	C20H34O2	305.2482	306.2559	305.2486	-1.24	2.6	[M-H]-
16	FA 20:5	FA 20:5	C20H30O2	301.2164	302.2246	301.2173	-3.09	1.6	[M-H]-
17	FA 22:3	FA 22:3	C22H38O2	333.2800	334.2872	333.2799	0.39	3.3	[M-H]-
18	FA 22:4	FA 22:4	C22H36O2	331.2642	332.2715	331.2643	-0.43	2.7	[M-H]-
19	FA 22:5	FA 22:5	C22H34O2	329.2486	330.2559	329.2486	0.14	2.1	[M-H]-
20	FA 22:6	FA 22:6	C22H32O2	327.2317	328.2402	327.2330	-4.10	1.9	[M-H]-
21	LPC 18:0	LPC 18:0	C26H54NO7P	524.3718	523.3638	524.3711	1.33	1.8	[M+H]+
22	LPC 18:2	LPC 18:2	C26H50NO7P	564.3304	519.3325	564.3307	-0.57	1.0	[M+HCOO]-
23	LPC 20:4	LPC 20:4	C28H50NO7P	588.3303	543.3325	588.3307	-0.73	1.0	[M+HCOO]-
24	LPE 18:2	LPE 18:2	C23H44NO7P	476.2781	477.2855	476.2783	-0.34	1.1	[M-H]-
25	PC 32:0	PC 16:0_16:0	C40H80NO8P	734.5721	733.5622	734.5694	3.68	8.0	[M+H]+
26	PC 32:1	PC 16:0_16:1	C40H78NO8P	732.5473	731.5465	732.5538	-8.85	6.3	[M+H]+
27	PC 34:2	PC 16:0_18:2	C42H80NO8P	802.5575	757.5622	802.5604	-3.61	6.7	[M+HCOO]-
28	PC 36:2	PC 18:0_18:2	C44H84NO8P	830.5889	785.5935	830.5917	-3.43	8.8	[M+HCOO]-
29	PC 36:4	PC 18:2_18:2	C44H80NO8P	826.5625	781.5622	826.5604	2.52	5.6	[M+HCOO]-
30	PC 36:5	PC 18:2_18:3	C44H78NO8P	824.5503	779.5465	824.5447	6.83	5.3	[M+HCOO]-
31	PC 38:5	PC 18:0_20:5<br>PC 16:0_22:5	C46H82NO8P	808.5861	807.5778	808.5851	1.29	7.1	[M+H]+
32	PC 40:5	PC 18:0_22:5	C48H86NO8P	836.6210	835.6091	836.6164	5.47	8.6	[M+H]+
33	PC 40:6	PC 18:0_22:6	C48H84NO8P	834.5973	833.5935	834.6007	-4.12	7.9	[M+H]+
34	PC 40:8	PC 18:2_22:6	C48H80NO8P	830.5625	829.5621	830.5694	-8.30	5.0	[M+H]+
35	PE 34:2	PE 16:0_18:2	C39H74NO8P	714.5114	715.5152	714.5079	4.86	7.2	[M-H]-
36	PE 36:2	PE 18:0_18:2	C41H78NO8P	742.5405	743.5465	742.5392	1.82	9.6	[M-H]-
37	PE 36:3	PE 18:1_18:2	C41H76NO8P	740.5297	741.5309	740.5236	8.20	7.4	[M-H]-
38	PE 36:4	PE 16:0_20:4	C41H74NO8P	740.5168	739.5152	740.5225	-7.68	7.0	[M+H]+
39	PE 36:5	PE 16:0_20:5	C41H72NO8P	736.4925	737.4996	736.4923	0.30	5.7	[M-H]-
40	PE 38:5	PE 18:0_20:5	C43H76NO8P	766.5408	765.5309	766.5381	3.54	7.6	[M+H]+
41	PE 38:6	PE 16:0_22:6	C43H74NO8P	762.5050	763.5152	762.5079	-3.80	6.5	[M-H]-
42	PE 38:7	PE 16:1_22:6	C43H72NO8P	760.4950	761.4996	760.4923	3.55	5.0	[M-H]-
43	PE 40:6	PE 18:0_22:6	C45H78NO8P	790.5354	791.5465	790.5392	-4.84	8.5	[M-H]-

44	PE O-38:5	PE O-18:1_20:4	C43H78NO7P	750.5432	751.5516	750.5443	-1.53	10.4	[M-H]-
45	PI 34:2	PI 16:0_18:2	C43H79O13P	833.5206	834.5258	833.5186	2.43	4.8	[M-H]-
46	PI 38:5	PI 18:0_20:5	C47H81O13P	883.5341	884.5415	883.5342	-0.09	5.1	[M-H]-
47	PS 38:6	PS 16:0_22:6	C44H74NO10P	806.4998	807.5050	806.4978	2.47	4.6	[M-H]-
48	SM 40:1;O2	SM 40:1;O2	C45H91N2O6P	831.6553	786.6615	831.6597	-5.29	13.1	[M+HCOO]-
49	SM 41:1;O2	SM 41:1;O2	C46H93N2O6P	801.6883	800.6771	801.6844	4.86	13.4	[M+H]+
50	SM 42:1;O2	SM 42:1;O2	C47H95N2O6P	815.6974	814.6928	815.7000	-3.25	13.7	[M+H]+
51	TG 52:2	TG (16:0_18:0_18:2)<br>TG (16:1_18:0_18:1)	C55H102O6	876.8034	858.7676	876.8015	2.13	16.5	[M+NH$_4$]+
52	TG 56:8	TG (16:0_18:2_22:6)<br>TG (16:1_18:2_22:5)<br>TG (16:0_20:4_20:4)<br>TG(18:2_18:1_20:5)<br>TG(18:2_18:2_20:4)<br>TG (16:1_20:2_20:5)	C59H98O6	920.7700	902.7363	920.7702	-0.22	15.3	[M+NH$_4$]+
53	TG 56:9	TG (18:2_18:3_20:4)<br>TG (16:1_18:2_22:6)<br>TG (16:1_18:3_22:5)<br>TG (18:2_18:2_20:4)	C59H96O6	918.7516	900.7207	918.7545	-3.20	15.0	[M+NH$_4$]+
HFD_WLEX									
a/a	Bulk number	Annotations	Neutral Formula	Exact mass m/z	Monoisotopic mass	Monoisotopic mass adduct	D(ppm)	Rt min	Adduct
1	DG 36:2	DG 18:1_18:1	C39H72O5	638.5662	620.5380	638.5718	-8.72	13.6	[M+NH$_4$]+
2	DG 36:3	DG 18:1_18:2	C39H70O5	636.5591	618.5223	636.5561	4.71	11.8	[M+NH$_4$]+
3	DG 36:4	DG 18:2_18:2	C39H68O5	634.5395	616.5067	634.5405	-1.58	9.4	[M+NH$_4$]+
4	DG 38:2	DG 18:1_20:1	C41H76O5	666.5978	648.5692	666.6031	-7.94	13.8	[M+NH$_4$]+
5	DG 38:3	DG 18:2_20:1	C41H74O5	664.5830	646.5536	664.5874	-6.55	13.3	[M+NH$_4$]+
6	DG 38:4	DG 18:1_20:3	C41H72O5	662.5709	644.5379	662.5718	-1.31	12.6	[M+NH$_4$]+
7	DG 40:8	DG 18:2_22:6	C43H68O5	682.5357	664.5066	682.5405	-6.97	8.1	[M+NH$_4$]+
8	FA 16:0	FA 16:0	C16H32O2	255.2338	256.2402	255.2330	3.13	2.7	[M-H]-
9	FA 18:2	FA 18:2	C18H32O2	279.2335	280.2402	279.2330	1.79	2.2	[M-H]-

10	FA 18:3	FA 18:3	C18H30O2	277.2171	278.2246	277.2173	-0.74	1.7	[M-H]-
11	FA 18:4	FA 18:4	C18H28O2	275.2012	276.2089	275.2017	-1.75	1.4	[M-H]-
12	FA 20:1	FA 20:1	C20H38O2	309.2801	310.2872	309.2799	0.80	3.7	[M-H]-
13	FA 20:2	FA 20:2	C20H36O2	307.2638	308.2715	307.2643	-1.65	3.0	[M-H]-
14	FA 20:3	FA 20:3	C20H34O2	305.2482	306.2559	305.2486	-1.24	2.6	[M-H]-
15	FA 20:5	FA 20:5	C20H30O2	301.2164	302.2246	301.2173	-3.09	1.6	[M-H]-
16	FA 22:3	FA 22:3	C22H38O2	333.2800	334.2872	333.2799	0.39	3.3	[M-H]-
17	FA 22:4	FA 22:4	C22H36O2	331.2642	332.2715	331.2643	-0.43	2.7	[M-H]-
18	FA 22:5	FA 22:5	C22H34O2	329.2486	330.2559	329.2486	0.14	2.1	[M-H]-
19	FA 22:6	FA 22:6	C22H32O2	327.2317	328.2402	327.2330	-4.10	1.9	[M-H]-
20	LPC 18:2	LPC 18:2	C26H50NO7P	564.3304	519.3325	564.3307	-0.57	1.0	[M+HCOO]-
21	LPE 18:2	LPE 18:2	C23H44NO7P	476.2781	477.2855	476.2783	-0.34	1.1	[M-H]-
22	PC 34:2	PC 16:0_18:2	C42H80NO8P	802.5575	757.5622	802.5604	-3.61	6.7	[M+HCOO]-
23	PC 36:2	PC 18:0_18:2	C44H84NO8P	830.5889	785.5935	830.5917	-3.43	8.8	[M+HCOO]-
24	PC 36:3	PC 18:1_18:2	C44H82NO8P	828.5724	783.5778	828.5760	-4.34	6.8	[M+HCOO]-
25	PC 36:3	PC 18:0_18:3	C44H82NO8P	784.5861	783.5778	784.5851	1.26	7.7	[M+H]+
26	PC 36:4	PC 18:2_18:2<br>PC 16:0_20:4	C44H80NO8P	826.5625	781.5622	826.5604	2.52	5.5-<br>6.4	[M+HCOO]-
27	PC 36:5	PC 18:2_18:3	C44H78NO8P	824.5503	779.5465	824.5447	6.83	5.3	[M+HCOO]-
28	PC 36:5	PC 16:0_20:5	C44H78NO8P	780.5514	779.5465	780.5538	-3.09	5.4	[M+H]+
29	PC 38:3	PC 18:0_20:3	C46H86NO8P	812.6186	811.6091	812.6164	2.71	9.5	[M+H]+
30	PC 38:5	PC 18:1_20:4	C43H76NO8P	808.5868	807.5778	808.5851	2.10	6.6	[M+H]+
31	PC 40:5	PC 18:0_22:5	C48H86NO8P	836.6210	835.6091	836.6164	5.47	8.6	[M+H]+
32	PC 40:8	PC 18:2_22:6	C48H80NO8P	830.5625	829.5621	830.5694	-8.30	5.0	[M+H]+
33	PE 34:2	PE 16:0_18:2	C39H74NO8P	714.5114	715.5152	714.5079	4.86	7.2	[M-H]-
34	PE 36:2	PE 18:0_18:2	C41H78NO8P	742.5405	743.5465	742.5392	1.82	9.6	[M-H]-
35	PE 36:3	PE 18:1_18:2	C41H76NO8P	740.5297	741.5309	740.5236	8.20	7.4	[M-H]-
36	PE 36:4	PE 16:0_20:4	C41H74NO8P	740.5168	739.5152	740.5225	-7.68	7.0	[M+H]+
37	PE 36:5	PE 16:0_20:5	C41H72NO8P	736.4925	737.4996	736.4923	0.30	5.7	[M-H]-
38	PE 38:5	PE 18:0_20:5	C43H76NO8P	766.5408	765.5309	766.5381	3.54	7.6	[M+H]+
39	PE 38:6	PE 16:0_22:6	C43H74NO8P	762.5050	763.5152	762.5079	-3.80	6.5	[M-H]-

40	PE 38:7	PE 16:1_22:6	C43H72NO8P	760.4950	761.4996	760.4923	3.55	5.0	[M-H]-
41	PE 40:6	PE 18:0_22:6	C45H78NO8P	790.5351	791.5465	790.5392	-5.19	8.5	[M-H]-
42	PE O-38:5	PE O-18:1_20:4	C43H78NO7P	750.5432	751.5516	750.5443	-1.53	10.4	[M-H]-
43	PI 34:2	PI 16:0_18:2	C43H79O13P	833.5206	834.5258	833.5186	2.43	4.8	[M-H]-
44	PI 38:5	PI 18:0_20:5	C47H81O13P	883.5341	884.5415	883.5342	-0.09	5.1	[M-H]-
45	SM 41:1;O2	SM 41:1;O2	C46H93N2O6P	801.6883	800.6771	801.6844	4.86	13.4	[M+H]+
46	SM 42:1;O2	SM 42:1;O2	C47H95N2O6P	815.6974	814.6928	815.7000	-3.25	13.7	[M+H]+
47	TG 52:4	TG (16:0_18:1_18:3)<br>TG (16:0_16:1_20:3)<br>TG (14:0_18:1_20:3)<br>TG (16:0_18:2_18:2)<br>TG (16:1_16:1_20:2)	C55H98O6	872.7750	854.7363	872.7702	5.50	15.5	[M+NH$_4$]+
48	TG 54:6	TG (18:0_18:1_20:5)<br>TG (18:0_18:3_20:3)<br>TG (18:0_18:2_20:4)<br>TG (18:1_18:2_20:3)<br>TG (18:2_18:2_20:2)<br>TG (16:0_18:1_22:5)<br>TG (16:1_18:0_22:5)	C57H98O6	896.7690	878.7363	896.7702	-1.37	15.4	[M+NH$_4$]+
49	TG 56:8	TG (16:0_18:2_22:6)<br>TG (16:1_18:2_22:5)<br>TG (16:0_20:4_20:4)<br>TG(18:2_18:1_20:5)<br>TG(18:2_18:2_20:4)<br>TG (16:1_20:2_20:5)	C59H98O6	920.7700	902.7363	920.7702	-0.22	15.3	[M+NH$_4$]+
50	TG 56:9	TG (18:2_18:3_20:4)<br>TG (16:1_18:2_22:6)<br>TG (16:1_18:3_22:5)<br>TG (18:2_18:2_20:4)	C59H96O6	918.7516	900.7207	918.7545	-3.20	15.0	[M+NH$_4$]+

Supplementary Table S4. P values and Log2FC for all sums of fatty acids and indices studied in the comparisons HFD-ND, HFD-WL, HFD-WLEX.

Fatty acids	HFD-ND		HFD-WL		HFD-WLEX	
Ratio	P value	log2FC	P value	log2FC	P value	log2FC
SFA	2.26E-01	0.20	1.92E-01	0.19	**1.64E-03**	0.33
MUFA	1.35E-02	-0.34	1.63E-01	-0.28	1.00E+00	-0.05
PUFA	**1.12E-03**	0.69	3.84E-01	0.42	4.63E-02	0.60
D5-desaturase	**2.55E-04**	1.04	8.89E-02	0.7	1.51E-02	0.88
D6-desaturase	**1.25E-04**	-1.55	7.13E-01	-0.68	**3.05E-03**	-1.34
D9-desaturase (cC16:1/C16:0)	8.89E-02	-0.44	**5.80E-04**	-0.81	5.85E-01	-0.34
D9-desaturase (cC18:1/C18:0)	3.12E-02	-0.68	1.68E-02	-0.43	2.55E-02	-0.65
DNL	**6.92E-05**	-0.65	2.85E-01	-0.36	4.63E-02	-0.48
Elong	1.00E+00	0.16	6.16E-02	0.26	1.68E-02	0.32
N6/N3	**1.73E-05**	-1.18	1.77E-01	-0.76	4.20E-02	-0.86
EFA	**3.86E-04**	1.00	3.31E-01	0.61	**4.37E-03**	0.92
NEFA	2.26E-01	-0.17	1.00E+00	-0.12	1.00E+00	0.08

Supplementary Table S5. Summary of all statistically significant identified lipids in adipose tissue of mice for the three binary comparisons, HFD_ND, HFD_WL, HFD_WLex. Information is provided regarding the structural formula and fatty acid chains of the lipids, molecular structure, monoisotopic mass, detected derivatives, retention time, and mass accuracy.

HFD_ND									
a/a	Bulk number	Annotations	Neutral Formula	Exact mass m/z	Monoisotopic mass	Monoisotopic mass adduct	D(ppm)	Rt min	Adduct
1	TG 38:2	TG(4:0_16:0_18:2) TG (4:0_16:1_18:1)	C41H74O6	680.5824	662.54854	680.5829	-0.73466	9.3	[M+NH4]+
2	TG 40:3	TG (4:0_18:1_18:2)	C43H76O6	706.5979	688.56419	706.5985	-0.84914	9.3	[M+NH4]+
3	TG 50:4	TG(16:1_16:1_18:2) TG(16:0_16:1_18:3) TG(14:0_18:2_18:2) TG(14:1_18:1_18:2) TG(14:0_18:1_18:3)	C53H94O6	844.7343	826.7050	844.7389	-5.45	11.2	[M+NH4]+
4	TG 50:5	TG(14:0/18:2/18:3) TG(16:1/16:1/18:3) TG(14:1/18:1/18:3)	C53H92O6	842.7234	824.68939	842.7237	-0.35599	10.8	[M+NH4]+
5	TG 51:3	TG(16:1_17:1_18:1) TG(16:0_17:1_18:2) TG(16:1_17:0_18:2)	C54H98O6	860.7652	842.7363	860.7702	-5.81	12.0	[M+NH4]+
6	TG 52:1	TG(16:0_18:0_18:1)	C55H104O6	878.8119	860.7833	878.8171	-5.92	13.2	[M+NH4]+
7	TG 52:2	TG(16:0_18:1_18:1) TG(16:1_18:0_18:1) TG(16:0_18:0_18:2)	C55H102O6	876.7972	858.7676	876.8015	-4.90	12.7	[M+NH4]+
8	TG 52:4	TG(16:1_18:1_18:2) TG(16:0_18:2_18:2) TG(16:0_18:1_18:3) TG(16:0_18:1_18:3)	C55H98O6	872.7659	854.7363	872.7702	-4.93	11.8	[M+NH4]+

9	TG 52:5	TG(16:1_18:2_18:2)<br>TG(16:0_18:2_18:3)<br>TG(16:1_18:1_18:3)	C55H96O6	870.7499	852.7207	870.7545	-5.28	11.3	[M+NH4]+
10	TG 52:6	TG(16:1_18:2_18:3)<br>TG(16:0_18:3_18:3)	C55H94O6	868.7356	850.7050	868.7389	-3.80	10.9	[M+NH4]+
11	TG 53:4	TG(17:1_18:1_18:2)<br>TG(17:0_18:2_18:2)	C56H100O6	886.7804	868.7520	886.7858	-6.09	12.0	[M+NH4]+
12	TG 54:2	TG(16:0_18:0_18:1)<br>TG(16:0_18:0_18:1)	C57H106O6	904.8284	886.7989	904.8328	-4.86	13.2	[M+NH4]+
13	TG 54:3	TG(18:1_18:1_18:1)<br>TG(16:1_18:1_20:1)<br>TG(16:0_18:2_20:1)	C57H104O6	902.8127	884.7833	902.8171	-4.87	12.7	[M+NH4]+
14	TG 54:4	TG(18:1_18:1_18:2)<br>TG(18:0_18:2_18:2)	C57H102O6	900.7964	882.7676	900.8015	-5.66162	12.3	[M+NH4]+
15	TG 54:5	TG(18:1_18:2_18:2)<br>TG(18:1_18:1_18:3)<br>TG(18:1_18:1_18:3)	C57H100O6	898.7805	880.7520	898.7858	-5.90	11.8	[M+NH4]+
16	TG 54:6	TG(18:1_18:2_18:3)	C57H98O6	896.7651	878.7363	896.7702	-5.69	11.4	[M+NH4]+
17	TG 54:7	TG(18:2_18:2_18:3)<br>TG(18:1_18:2_18:4)<br>TG(18:1_18:3_18:3)	C57H96O6	894.7499	876.7207	894.7545	-5.14	11.0	[M+NH4]+
18	TG 56:2	TG(18:0_18:1_20:1)<br>TG(18:1_18:1_20:0)	C59H110O6	932.8570	914.8302	932.8641	-7.61	13.7	[M+NH4]+
19	TG 56:3	TG(18:0_18:1_20:2)<br>TG(18:0_18:2_20:1)<br>TG(18:1_18:2_20:0)<br>TG(18:1_18:1_20:1)	C59H108O6	930.8416	912.8146	930.8484	-7.30516	13.2	[M+NH4]+
20	TG 56:4	TG(18:1_18:2_20:1)<br>TG(18:1_18:1_20:2)<br>TG(18:0_18:2_20:2)<br>TG(18:2_18:2_20:0)	C59H106O6	928.8272	910.7989	928.8328	-6.03	12.8	[M+NH4]+

21	TG 56:5	TG(18:1_18:2_20:2) TG(18:1_18:1_20:3) TG(18:0_18:2_20:3) TG(16:0_18:1_22:4) TG(16:0_20:2_20:3) TG(18:2_18:2_20:1) TG(16:0_18:0_22:5) TG(16:0_18:2_22:3)	C59H104O6	926.8113	908.7833	926.8171	-6.26	11.8-12.3	$[M+NH_4]+$
22	TG 56:6	TG(16:0_18:2_22:4) TG (16:0_18:1_22:5) TG (18:1_18:2_20:3) TG (18:2_18:2_20:2)	C59H102O6	924.7939	906.7676	924.8015	-8.22	11.4	$[M+NH_4]+$
HFD_WL									
	**Bulk number**	**Annotations**	**Neutral Formula**	**Exact mass m/z**	**Monoisotopic mass**	**Monoisotopic mass adduct**	**D(ppm)**	**Rt min**	**Adduct**
1	TG 38:1	TG(4:0_16:0_18:1)	C41H76O6	682.5977	664.56419	682.5985	-1.17199	9.7	$[M+NH_4]+$
2	TG 38:2	TG(4:0_16:0_18:2) TG (4:0_16:1_18:1)	C41H74O6	680.5824	662.54854	680.5829	-0.73466	9.3	$[M+NH_4]+$
3	TG 40:1		C43H80O6	710.6294	692.59549	710.6298	-0.56288	9.7	$[M+NH_4]+$
4	TG 40:3	TG (4:0_18:1_18:2)	C43H76O6	706.5979	688.56419	706.5985	-0.84914	9.3	$[M+NH_4]+$
5	TG 42:2		C45H82O6	736.6445	718.61114	736.64551	-1.37108	9.7	$[M+NH_4]+$
HFD_WLEX									
**a/a**	**Bulk number**	**Annotations**	**Neutral Formula**	**Exact mass m/z**	**Monoisotopic mass**	**Monoisotopic mass adduct**	**D(ppm)**	**Rt min**	**Adduct**
4	TG 40:3	TG (4:0_18:1_18:2)	C43H76O6	706.5979	688.56419	706.5985	-0.84914	9.3	$[M+NH_4]+$
5	TG 42:2		C45H82O6	736.6445	718.61114	736.64551	-1.37108	9.7	$[M+NH_4]+$

Supplementary table S6. Description of the extractions performed on the mice liver and adipose tissue samples.

	Liver		Adipose tissue				
Mice	mg Liver	µL MTBE: MeOH (3:1 v/v)	mg AT	µL MeOH	µL $CHCl_3$:MeOH (7:1 v/v)	µL $CHCl_3$	µL H_2O
HDF_1	29.9	454	9.50	190	608	342	171
HDF_2	45.9	699	12.1	242	774	436	218
HDF_3	29.7	452	11.0	220	704	396	198
HDF_4	20.8	316	10.2	204	653	367	184
HDF_5	48.8	743	9.6	192	614	346	173
HDF_6	50.3	765	10.4	208	666	374	187
HDF_7	29.2	445	14.2	284	909	511	256
HFDEX_1	19.6	299	7.00	140	448	252	126
HFDEX_2	37.0	563	4.00	80	256	144	72
HFDEX_3	36.4	554	4.30	86	275	155	77
HFDEX_4	49.1	748	4.70	94	301	169	85
HFDEX_5	78.8	1200	3.90	78	250	140	70
HFDEX_6	36.5	555	12.7	254	813	457	229
HFDEX_7	52.5	799	9.70	194	621	349	175
ND_1	26.2	399	4.80	96	307	173	86
ND_2	27.4	417	6.30	126	403	227	113
ND_3	26.7	407	6.80	136	435	245	122
ND_4	22.5	342	12.5	250	800	450	225
ND_5	35.0	533	9.70	194	621	349	175
ND_6	24.0	365	6.30	126	403	227	113
ND_7	22.4	340	11.9	238	762	428	214
WL_1	41.1	625	2.50	50	160	90	45
WL_2	26.7	406	7.50	150	480	270	135
WL_3	40.6	618	5.60	112	358	202	101
WL_4	28.7	437	10.3	206	659	371	185
WL_5	27.1	413					
WL_6	15.1	230	8.00	160	512	288	144
WL_7	24.1	367	10.1	202	646	364	182
WLEX_1	28.3	430	12.5	250	800	450	225
WLEX_2	32.5	495	10.1	202	646	364	182
WLEX_3	18.3	278	10.4	208	666	374	187
WLEX_4	17.2	261	10.4	208	666	374	187
WLEX_5	16.6	253	8.60	172	550	310	155
WLEX_6	29.4	448	10.7	214	685	385	193
WLEX_7	34.0	518	8.90	178	570	320	160
